# Accuracy and Limitations of the Pair‐Selected Multilevel Approach for DLPNO Coupled Cluster: Extensive Benchmark for Closed‐Shell Organic Reactions

**DOI:** 10.1002/cphc.202500246

**Published:** 2025-11-18

**Authors:** Nalini Gurav, Nadim Ramez, Lukas Lampe, Johannes Neugebauer

**Affiliations:** ^1^ Theoretische Organische Chemie Organisch‐Chemisches Institut and Center for Multiscale Theory and Computation Universität Münster Corrensstraße 36 48149 Münster Germany

**Keywords:** local coupled cluster, multilevel coupled cluster, reaction barrier benchmark, reaction energy benchmark

## Abstract

Reliable approximations to coupled‐cluster (CC) methods are highly desirable for accurate yet efficient computations of barrier heights, reaction energies, and other molecular properties. Among these methods, domain‐based local pair natural orbital CC with singles, doubles, and perturbative triple excitations [DLPNO‐CCSD(T)] is widely used due to its formal linear scaling with the system size. However, since DLPNO‐CCSD(T) remains costly, the extension to multilevel (ML) variants becomes an obvious route. This strategy can be made even more economic with the pair‐selected ML ansatz [M. Bensberg and J. Neugebauer, J. Chem. Phys. **157**, 064102 (2022)] to DLPNO‐CCSD(T_0_) with a semi‐canonical (SC) perturbative triples correction. This ansatz uses an automatic partitioning of orbital pairs according to their contribution to the overall correlation energy change in a chemical reaction. Herein, the advantages of this approach are demonstrated for closed‐shell organic reactions of the BH9 test set. The errors are nearly always within chemical accuracy (4 kJ mol^−1^) along with a significant time benefit. In rare cases, larger errors are observed. These are analyzed by comparison of SC and iterative perturbative triples, of different ML thresholds, and of ML and single‐level schemes. A beneficial error cancelation between DLPNO and ML contributions is also observed in several cases.

## Graphical abstract

This graphic shows the error distribution for pericyclic reactions calculated with the multilevel approach under investigation and illustrates that outliers are critically analyzed using different approximations for the triples correction.

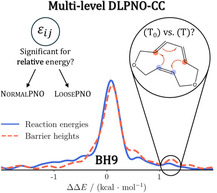

## 1 Introduction

One of the principal goals of computational quantum chemistry is the accurate calculation of energy profiles for chemical reactions. Of particular importance are barrier heights and reaction energies for reaction kinetics and thermodynamics, respectively. For single‐reference cases, coupled‐cluster (CC) methods^[^ ^1^, ^2^, ^3^ ^]^ generally provide accurate relative energies. More specifically, CC with singles, doubles, and perturbative triple excitations [CCSD(T)]^[^ ^4^ ^]^ has been established as the so‐called “gold‐standard” of quantum chemistry, because an accuracy of about 4 kJ mol^−1^ for relative energies, often termed “chemical accuracy”, is reached.^[^ ^5^ ^]^

The steep computational scaling with system size, for example, *O*(N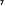) for CCSD(T), can be reduced through several approximations like the cluster‐in‐molecule approach,^[^ ^6^, ^7^ ^]^ fragmentation‐based schemes such as the molecular tailoring approach^[^ ^8^ ^]^ and divide‐and‐conquer methods^[^ ^9^ ^]^ as well as through embedding schemes^[^ ^10^ ^]^ combining, for example, density functional theory (DFT) and wave function methods using orbital localization.^[^ ^11^ ^]^ The most common approximate CC variants are local correlation approaches, especially the domain‐based local pair natural orbital CC (DLPNO‐CC) approach.^[^ ^12^, ^13^, ^14^, ^15^ ^]^ Such local correlation methods were originally motivated by Pulay and Saebø^[^ ^16^, ^17^, ^18^ ^]^ and exploit the locality of dynamic electron correlation^[^ ^19^ ^]^ based on pair natural orbitals (PNOs).^[^ ^20^ ^]^ Significant developments in local correlation methods have been driven through the works by Werner,^[^ ^19^, ^21^ ^]^ Schütz,^[^ ^22^, ^23^ ^]^ Tew,^[^ ^24^, ^25^ ^]^ Head‐Gordon,^[^ ^26^, ^27^ ^]^ Nagy,^[^ ^28^, ^29^ ^]^ and in the context of DLPNO‐CC by Neese and coworkers.^[^ ^12^, ^13^, ^14^, ^20^, ^30^, ^31^, ^32^, ^33^ ^]^

For localized chemical processes in large systems, it may not be required to describe the whole system at the level which is associated with the desired target accuracy, since errors made in the treatment of “spectator fragments” may systematically cancel out when computing energy differences. Then, these local correlation approaches can be extended to multilevel (ML) variants^[^ ^34^ ^]^ for a target‐oriented use of computational resources. In ML calculations,^[^ ^35^ ^]^ the system is partitioned into multiple regions/fragments which are treated at different degrees of approximation. For instance, local CC methods are often combined with computationally less expensive local second‐order Møller–Plesset perturbation theory^[^ ^36^, ^37^, ^38^ ^]^ or with local CC using less tight settings.^[^ ^34^ ^]^ The partitioning in these ML calculations is mostly carried out in terms of the occupied orbital spaces. Since the partitioning of occupied orbital spaces is crucial for the accuracy and computational efficiency, it becomes desirable to identify distinct criteria that come in hand with a targeted accuracy and computational efficiency. There are several partitioning schemes commonly involved in ML local correlation approaches that are briefly discussed in the following.

An intuitive partitioning scheme follows from a manual selection of atoms that are involved in the reaction. The occupied orbitals are then classified based on the atoms they are localized on.^[^ ^34^, ^37^ ^]^ However, targeting a specific accuracy is then not a priori possible. This can be achieved with automatic, quantifiable partitionings such as the direct orbital selection (DOS) approach,^[^ ^39^ ^]^ developed earlier in our lab. The DOS was initially developed to automatically select orbital‐sets within a DFT embedding framework, and was applied, for example, in the context of DFT‐in‐DFT embedding^[^ ^39^ ^]^ as well as in wavefunction‐in‐DFT embedding for transition‐metal coordination reactions.^[^ ^40^, ^41^, ^42^ ^]^ As demonstrated in the work done by Bensberg and Neugebauer,^[^ ^40^ ^]^ the errors obtained with DOS are, in general, smaller than those obtained with conventional manual selection schemes for the same numbers of selected orbitals. In turn, an intuitive manual selection in general requires more active orbitals to reach the same target accuracy. To give an example, to obtain the target accuracy of about 4 kJ mol^−1^ for the Stone–Wales‐defect formation with B2PLYP‐in‐BLYP embedding, about 50 active orbitals were needed with DOS, while more than 100 active orbitals were required with the manual selection in the work done by Bensberg and Neugebauer.^[^ ^40^ ^]^ The DOS approach was then combined with the ML DLPNO‐CC approach^[^ ^34^ ^]^ and its efficiency was demonstrated for predicting reaction energies employing ML DLPNO‐CCSD(T_0_)^[^ ^13^ ^]^ using a semi‐canonical (SC) triples correction.^[^ ^43^ ^]^ As it was shown that important contributions of orbital pairs can be sorted out with the DOS,^[^ ^42^ ^]^ a superior strategy is an orbital‐pair‐based selection, referred to as pair‐selected ML approach.^[^ ^44^ ^]^ The DOS algorithm can be used as a starting point for the pair selection as it also establishes a mapping of occupied orbital‐sets along a reaction pathway based on orbital‐wise partial charges and orbital‐wise kinetic energies.^[^ ^39^ ^]^ This enables approximating correlation energy changes of orbital‐pair sets to identify significant contributions that can be treated with tighter settings. Previously, this pair‐selected ML approach has been applied to DLPNO‐CCSD(T_0_)^[^ ^44^ ^]^ with promising accuracy for several benchmark sets, including interaction energies of weakly interacting complexes (6–34 atoms, benchmark set from the work done by Rezác et al.)^[^ ^45^ ^]^ reaction energies of large transition‐metal complexes (42–174 atoms, from the work done by Weymuth et al.)^[^ ^46^ ^]^ and reaction energies of smaller systems (11–30 atoms, from the work done by Friedrich and Hänchen).^[^ ^47^ ^]^ For the S66×8 benchmark set (the work done by Bensberg and Neugebauer),^[^ ^45^ ^]^ the pair‐selected DLPNO‐CCSD(T_0_) approach reproduces the single‐level (SL) results with mean absolute deviations (MADs) of 0.72 kJ mol^−1^ or less, depending on the reactions and maximum deviations of up to 4.76 kJ mol^−1^ or less. Overall, the accuracy is comparable to that obtained for the other benchmark sets,^[^ ^46^, ^47^ ^]^ confirming that the method reliably describes both covalent and noncovalent interactions within the approximations by DLPNO‐CCSD(T_0_) already introduced.

In recent work,^[^ ^48^ ^]^ an alternative strategy to the original DOS‐based pair‐selected ML DLPNO approach was proposed. Instead of the DOS *orbital‐set* mapping, which may leave a fraction of orbitals unmapped, this scheme introduced a localization‐based bijective orbital mapping relying on partial charges, thereby yielding complete orbital correspondences. In addition, the DLPNO thresholds were decoupled such that pair cutoffs and PNO truncation parameters could be adjusted independently to control pair and domain approximations. While these refinements improved the performance relative to the original DOS approach, they also introduced severe problems for some BH9 reactions when diffuse basis functions were employed. In such cases, large errors originating from the SC triples correction were observed both for the ML and the conventional DLPNO‐CCSD(T_0_) using loose thresholds. Whether such deficiencies also persist for full (iterative) triples has so far remained unexplored.

In this work, we therefore revisit the original DOS‐based pair‐selected ML approach and apply it to the BH9 benchmark set. To provoke potential error sources, we deliberately include diffuse basis functions and use NormalPNO thresholds. Moreover, we extend the approach to iterative triples [DLPNO‐CCSD(T_1_)],^[^ ^33^ ^]^ allowing us to directly compare the DOS‐based and localization‐based schemes under consistent conditions. Previous studies have shown that the DLPNO‐CCSD(T_0_) variant may introduce significant errors.^[^ ^33^, ^49^ ^]^ Then, the iterative triples correction combined with DOS‐based pair‐selection from the work done by Bensberg and Neugebauer^[^ ^44^ ^]^ is compared with the pair‐selection method from the work done by Lampe and Neugebauer^[^ ^48^ ^]^ called multilevel2 (ML2) in the following including the iterative triples. Note that in the literature this full triples correction is sometimes denoted DLPNO‐(T_1_), in line with the notation for the SC correction DLPNO‐(T_0_), and we adopt this notation here. Finally, we benchmark the method against canonical CCSD(T) results from the work done by Lampe and Neugebauer^[^ ^48^ ^]^ to assess its accuracy, and we apply it to a 135‐atom model system^[^ ^50^ ^]^ to evaluate both accuracy and computational efficiency.

## 2 Theory

The pair‐selected ML approach requires the construction of maps between localized occupied orbitals that can be carried out through the DOS method.^[^ ^39^, ^40^ ^]^ The DOS is an efficient black‐box tool for localized orbital analysis along a reaction path, and incidentally, constructs orbital‐set maps and categorizes them into “mappable” and “unmappable”. A detailed explanation of the DOS can be found in the refs. [39, 40] and [44]. We only briefly discuss the concepts in the following subsections.

### 2.1 Orbital‐Set‐Map Construction in the DOS

In the DOS algorithm, occupied orbitals of two structures *L* and *K* are mapped by comparing orbital kinetic energies $t_{\text{i}}$ and orbital‐wise partial charges $q_{\text{i}}$. For a given orbital $\psi_{\text{iL}}$ of structure *L*, a candidate orbital $\psi_{\text{iK}}$ in structure *K* is considered a match if

(1) $$|t_{iL}-t_{jK}|<\tau_{\text{kin}}$$

(2) $$\sum_{a}|q_{\text{iL}}^{\text{a}}-q_{\text{jK}}^{\text{a}}|<\tau_{\text{loc}}$$

where $t_{\text{iL}}$ is the orbital kinetic energy and $q_{\text{iL}}^{\text{a}}$ is the partial charge of orbital iL on atom (or atomic shell) a. The thresholds $\tau_{\text{kin}}$ and $\tau_{\text{loc}}$ ^[^ ^40^, ^44^ ^]^ control the similarity criteria for the orbitals. The two conditions above yield surjective maps from orbitals of *L* to sets of orbitals of *K* and vice versa, for example,

(3) $$\psi_{\text{iL}}\to {\left\{\psi_{\text{jK}}\right\}}_{\text{iL}}$$

(4) $$\psi_{\text{jK}}\to {\left\{\psi_{\text{jL}}\right\}}_{\text{jK}}$$

**Figure 1:**
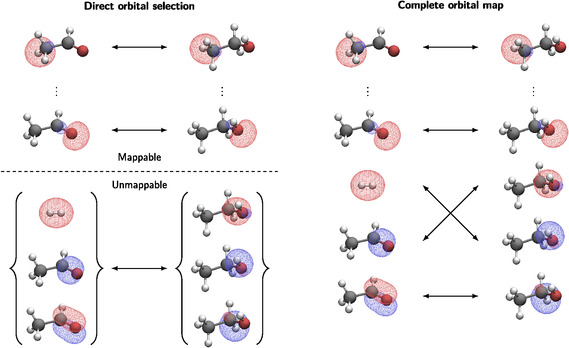
Localized valence orbitals are shown for the hydrogenation of acetaldehyde to visualize orbital‐set maps from the DOS approach as used in ref. [44] (left) and complete orbital maps as used in ref. [48] (right).

The orbital‐sets are assigned bijectively (one‐to‐one) by matching sets of equal cardinality (size of the set), to ensure consistent orbital correspondences along the reaction pathway

(5) $${\left\{\psi_{\text{jK}}\right\}}_{\text{iL}}=\{\psi_{\text{jK}}\}\;\forall (iL)\;\text{with}\;\psi_{\text{iL}}\in \{\psi_{\text{jL}}\}$$

(6) $${\left\{\psi_{jL}\right\}}_{\text{iK}}=\{\psi_{\text{jL}}\}\;\forall (iK)\;\text{with}\;\psi_{\text{iK}}\in \{\psi_{\text{jK}}\}$$

where ${\left\{\psi_{jK}\right\}}_{jL}$ and ${\left\{\psi_{\text{jL}}\right\}}_{\text{iK}}$ are the sets from the surjective maps (Equation (3) and (4)). This mapping procedure can be extended to more than two structures to include, for example, reactants, transition state, and products. In addition, there may remain occupied orbitals which are not a member of any mapped orbital‐set. Such orbitals are assigned to a set of unmappable orbitals ${\left\{\psi_{iL}\right\}}^{A}$ which naturally has the same cardinality for every structure on the reaction coordinate. The sizes of these orbital‐sets (or the number of mappable and unmappable orbitals) directly depends on the DOS thresholds $\tau_{\text{kin}}$ and $\tau_{\text{loc}}$. Consequently, all occupied orbitals are contained in the union of all orbitals, including the unmappable ones. In practice, a series of DOS thresholds, typically three (also discussed in the Experimental Section), is applied to ensure that the size of the orbital sets in the maps is small, where the most loose DOS threshold determines the sets of unmappable orbitals. The construction of these maps is illustrated in **Figure** **1**, where the orbital construction for a hydrogenation of acetaldehyde is visualized. On the left side, the DOS‐based pair‐selection approach^[^ ^44^ ^]^ (ML) is shown, illustrating the separation of mappable and unmappable orbitals. The DOS approach provides a mapping of different orbital‐sets,

(7) $${\left\{\psi_{\text{iL}}\right\}}_{\text{I}}↔{\left\{\psi_{\text{iK}}\right\}}_{\text{I}}$$

where the orbital‐set I can describe a mappable or unmappable orbital‐set for structures *L* and *K*. On the right side, the complete orbital‐map construction from the work done by Lampe and Neugebauer^[^ ^48^ ^]^ (ML2) is shown, in which all orbitals are assigned.

### 2.2 Orbital‐Pair Set Prescreening and Triples Correction in the Pair‐Selected ML DLPNO‐CC Ansatz

The pair‐selected ML approach is based on a decomposition of the relative correlation energy into relative pair correlation energies. In the DLPNO‐CCSD approach, the correlation energy of a closed‐shell system *K* can be expressed in terms of a sum over pair energies $\varepsilon_{\text{K}}^{\text{ij}}$

(8) $$E_{\text{K}}^{\text{C,DLPNO}-\text{CCSD}}=\sum_{\text{i}\leq \text{j}}\varepsilon_{\text{K}}^{\text{ij}}.$$

These pair energies can be grouped into pair contributions $e_{\text{K}}^{\text{IJ}}$ of orbital‐sets ${\left\{\psi_{iK}\right\}}_{I}$ and ${\left\{\psi_{jK}\right\}}_{J}$ as

(9) $$e_{\text{K}}^{\text{IJ}}=\underset{\begin{array}{c} \text{with}\;\psi_{\text{iK}}\in {\left\{\psi_{\text{iK}}\right\}}_{\text{I}}\\ \text{and}\;\psi_{\text{jK}}\in {\left\{\psi_{\text{jK}}\right\}}_{\text{J}} \end{array}}{\underbrace{\sum_{\text{i}\leq \text{j}}}}\varepsilon_{\text{K}}^{\text{ij}}.$$

By applying the orbital‐set map as introduced above, the relative correlation energy becomes a sum over increments $\Delta e_{\text{KL}}^{\text{IJ}}$ associated with relative changes of orbital‐pair sets

(10) $$\Delta E_{\text{KL}}^{\text{C,DLPNO}-\text{CCSD}}=\sum_{\text{I}\leq \text{J}}(e_{\text{K}}^{\text{IJ}}-e_{\text{L}}^{\text{IJ}})=\sum_{\text{I}\leq \text{J}}\Delta e_{\text{KL}}^{\text{IJ}},$$

where the enumeration indices *I* and *J* correspond to orbital‐sets which are mapped onto each other between the two structures. In practice, the contributions $\Delta e_{\text{KL}}^{\text{IJ}}$ are obtained by SC MP2 with LoosePNO settings and then are compared against pair selection thresholds $\tau_{\Delta }$ to categorize orbital pairs into different accuracy levels of the DLPNO settings. Orbital pairs that contribute significantly (above a threshold $\tau_{\Delta }$) are treated with high accuracy, while more approximate settings are used for others. The generalized pair‐selection criterion for two or more molecular structures can be stated as follows

(11) $$|\Delta e_{\text{max}}^{\text{IJ}}|>\tau_{\Delta }\;\text{with}\;|\Delta e_{\text{max}}^{\text{IJ}}|=\max_{KL}|\Delta e_{\text{KL}}^{\text{IJ}}|.$$

The orbital pairs within orbital‐pair sets described with less accurate DLPNO settings are more likely to be excluded from the amplitude optimizations by the pair cutoff $\tau_{\text{CutPairs}}$ ^[^ ^12^ ^]^ compared to more accurate settings. Therefore, as described in the work done by Bensberg and Neugebauer,^[^ ^44^ ^]^ to avoid unstable amplitude optimizations the pair cutoff for an orbital pair ij is constrained to the minimum value that can be found for any orbital‐pair set IJ which includes *i* or *j*.

For the SC triples correction, the calculation of each triple ijk depends on the previous prescreening of orbital‐pair sets to which the three pairs ij, ik, and jk correspond as explained in the work done by Bensberg and Neugebauer:^[^ ^44^ ^]^ 1) only triples ijk are considered with at least two of the three pairs ij, ik, and jk being categorized as strong pairs according to $\tau_{\text{CutPairs}}$; 2) the amplitudes from DLPNO‐CCSD enter the expression of the triple energy. In case of the iterative triples correction, only these selected triples are then further processed according to the work done by Bensberg and Neugebauer.^[^ ^39^ ^]^ Consequently, both the SC and the iterative triples correction benefit—in terms of their computational cost—from the reduced numbers of orbital triples. A detailed workflow and an example input, including all features from a ML DLPNO‐CCSD(T_1_) calculation are shown in the Supporting Information. The DLPNO‐CCSD(T_1_) and DLPNO‐CCSD(T_0_) corrections are both calculated according to the work done by Guo et al.^[^ ^33^ ^]^

## 3 Experimental Section

We investigate five sets of chemical reactions from the BH9 dataset,^[^ ^51^, ^52^ ^]^ covering pericyclic, hydride transfer, proton transfer, and nucleophilic addition/substitution reactions. **Table** **1** lists these sets of reactions, each with a prototypical example. We limit ourselves to the closed‐shell cases, as our implementation is currently not yet generalized to open‐shell treatments. In total, we analyzed 233 closed‐shell organic reactions with system sizes ranging from 12 to 71 atoms, ensuring sufficient complexity to assess the accuracy of the employed method. To give an indication of the system size distribution, we mentioned that reactions investigated include 29 reactions with 10–20 atoms, 149 reactions with 21–50 atoms, and 55 reactions with more than 50 atoms. The BH9 dataset consists of reactions that involve typical main‐group elements such as H, C, N, O, F, P, S, Cl, B, and Si.

**Table 1:** Closed‐shell reaction sets from the BH9 benchmark set. The roman indices in parenthesis are adopted from ref. [51].

Reaction type	Number of reactions	Example reaction
Pericyclic (II)	140	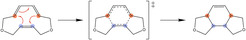
Hydride transfer (V)	42	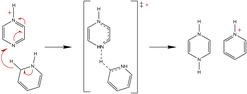
Proton transfer (VII)	10	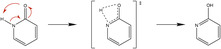
Nucleophilic substitution (VIII)	15	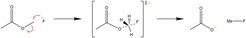
Nucleophilic addition (IX)	26	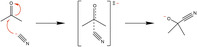

The Cartesian coordinates of all structures were adopted from the work done by Prasad et al.^[^ ^51^ ^]^ Atomic coordinates in the geometry files of transition state and product structure were reordered according to their correspondence to the atoms of the reactant. This step is necessary for the DOS scheme^[^ ^39^ ^]^ and the orbital alignment procedure,^[^ ^40^ ^]^ which both require a one‐to‐one correspondence between atoms in successive structures. Atom maps were initially generated using a preliminary version of the algorithm proposed in the work done by Lampe and Neugebauer^[^ ^48^ ^]^ but each reaction was manually verified or corrected where necessary. Additionally, a test set of a decarboxylation reaction is used, to compare timings for systems over 100 atoms. The geometries for the model III (135 atoms) from the work done by Liao et al.^[^ ^50^ ^]^ are used with def2‐TZVP as basis set. In this calculation, the timings and energy errors for ML and SL DLPNO‐CCSD(T_1_/T_0_) are investigated on an AMD EPYC node (see below for details).

All calculations were performed using a development version of the quantum chemistry program Serenity.^[^ ^53^ ^]^ Computations were carried out on two types of computing nodes: Intel(R) Xeon(R) Silver 4216 CPU @ 2.10 GHz with 32 cores and dual AMD EPYC 7643 48‐core processors with 96 cores. For a comparison of timings, the same computational nodes were used for reference and ML calculations of the same reaction.

For each system, Hartree–Fock orbitals were computed with Dunning's augmented correlation‐consistent polarized valence triple‐*ζ* basis set (aug‐cc‐pVTZ)^[^ ^54^ ^]^ and localized using the intrinsic bond orbital (IBO) approach.^[^ ^55^ ^]^ These localized orbitals were used for reference calculations, also referred to as SL calculations, using DLPNO‐CCSD(T_0_), and also DLPNO‐CCSD(T_1_) for a few cases, with NormalPNO settings and frozen‐core approximation for inactive core orbitals. The NormalPNO setting was chosen for its balance between computational efficiency and accuracy, compared to the more demanding TightPNO settings. For ML calculations, the localization procedure additionally involves aligning^[^ ^40^ ^]^ the orbitals of transition state and product structures with respect to those of the reactant structure prior to the IBO localization. The aligned and localized orbitals are then mapped onto each other with the DOS scheme. In this work, the pair‐selection ansatz is employed in a black‐box manner. Thus, a spread threshold triple of $\tau_{\text{kin}}/E_{h}=(1\cdot 10^{-1},5\cdot 10^{-3},1\cdot 10^{-4})$ adopted from previous studies^[^ ^44^ ^]^ is utilized, ensuring compact orbital subsets. The corresponding localization thresholds $\tau_{\text{loc}}$ are determined as described in the work done by Bensberg and Neugebauer.^[^ ^40^, ^44^ ^]^ All orbitals that are mappable with the tightest threshold $\tau_{\text{kin}}=1\cdot 10^{-4}\;E_{h}$ are removed from the correlation treatment that is explained in the following. The pair energies in Equation (8) were approximated with LoosePNO SC‐MP2. According to a previous recommendation,^[^ ^44^ ^]^ a pair selection threshold $\tau_{\Delta }=1\cdot 10^{-3}\;E_{h}$ is chosen in general and, only for a few pericyclic reactions, a threshold of $\tau_{\Delta }=1\cdot 10^{-4}\;E_{h}$ was applied. These particular cases are recalculated with a tighter threshold because of errors in relative energies that exceed 4 kJ mol^−1^ using $\tau_{\Delta }=1\cdot 10^{-3}\;E_{h}$. Applying Equation (11) for the given threshold, the orbital‐pair sets are partitioned into two subsets: one containing significant orbital‐pair sets and the other one comprising nonsignificant ones. The subset of significant orbital pair sets is then treated with NormalPNO, while the other one is treated with LoosePNO. Subsequently, a DLPNO‐CCSD(T_0_) or DLPNO‐CCSD(T_1_) calculation can be performed based on these orbital‐pair specific settings. An overview of an input data and the important steps of such a pair‐selected ML calculation is given in the Figure S3, Supporting Information. In case of ML2 calculations, complete orbital maps were constructed following the procedure in the work done by Lampe and Neugebauer.^[^ ^48^ ^]^ Subsequently, the modified pair selection scheme was conducted using three ML thresholds: $\tau_{\text{CutPNO}}^{\Delta }=10^{-3}\;E_{h}$, $\tau_{\text{CutPairs}}^{\Delta }=10^{-5}\;E_{h}$, and $\tau_{\text{CutTriples}}^{\Delta }=10^{-5}\;E_{h}$. This threshold combination was recommended for a target accuracy of NormalPNO^[^ ^48^ ^]^ and can be found in Table S11, Supporting Information.

In the Results section, canonical CCSD(T) results for the proton transfer reactions are compared to ML and SL DLPNO‐CCSD(T_1_/T_0_) calculations, with a def2‐TZVP^[^ ^56^ ^]^ basis set and NormalPNO settings. The CCSD(T) results are taken from the work done by Lampe and Neugebauer.^[^ ^48^ ^]^

To assess the performance of the pair‐selected ML ansatz, we evaluate the errors in relative energies by comparing ML and SL or reference calculations. The relative energy error of reaction *i* is defined as

(12) $$\Delta \Delta E_{\text{i}}=\Delta E_{\text{i}}^{\text{multi}}-\Delta E_{\text{i}}^{\text{ref}}$$

where $\Delta E_{i}^{\text{multi}}$ represents the relative energy obtained from the ML calculation, and $\Delta E_{\text{i}}^{\text{ref}}$ corresponds to the reference calculation. We analyze systematic trends in relative energy errors, in terms of MAD and maximum absolute error (MAX), defined as

(13) $$\text{MAD}=\frac{1}{N}\sum_{i}^{N}|\Delta \Delta E_{i}|$$

(14) $$\text{MAX}=\max_{\text{i}}|\Delta \Delta E_{\text{i}}|$$

for a set of *N* relative energy differences, and the standard deviation (SD), defined as

(15) $$\text{SD}=\sqrt{\frac{1}{N}\sum_{\text{i}}^{\text{N}}{\left(|\Delta \Delta E_{\text{i}}|-\text{MAD}\right)}^{2}}$$

## 4 Results and Discussion

### 4.1 Error Distributions for Different Reaction Types

**Figure 2:**
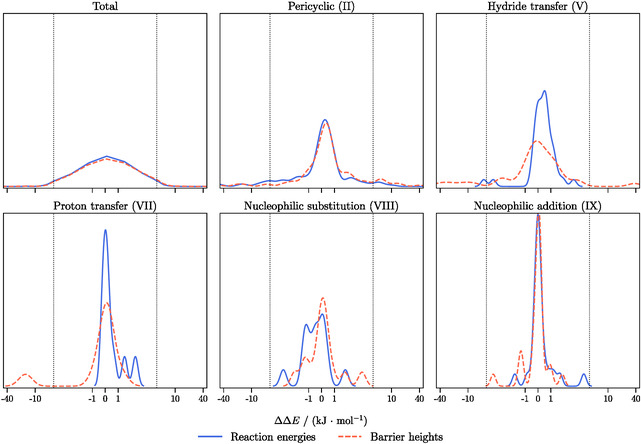
Error distributions in kJ mol^−1^ for reaction energies and barrier heights for different subsets of the BH9 benchmark set calculated with pair‐selected ML DLPNO‐CCSD(T_0_) with $\tau_{\Delta }=10^{-3}\;E_{h}$ with respect to NormalPNO DLPNO‐CCSD(T_0_ $T_{0}$). The aug‐cc‐pVTZ basis set is used. Note the logarithmic scale on the x‐axis.

The error distributions with respect to NormalPNO DLPNO‐CCSD(T_0_) calculated for all closed‐shell reactions of the BH9 set are presented in **Figure** **2**. These distributions were determined for reaction energies (blue) and barrier heights (red) across different reaction types, including pericyclic (II), hydride transfer (V), proton transfer (VII), nucleophilic substitution (VIII), and nucleophilic addition (IX).

The total error distribution is centered around 0 kJ mol^−1^ for reaction energies and barrier heights with most of the errors distributed between −4 and 4 kJ mol^−1^. For nucleophilic substitution and addition reactions, all errors are distributed between −4 and 4 kJ mol^−1^. However, minor peaks outside this interval are observed for pericyclic, proton transfer, and hydride transfer reactions. Especially for pericyclic and hydride transfer reactions, these are small and barely noticeable, likely representing individual outliers. Since these reaction sets contain a significantly larger number of test molecules with (140 and 42, respectively, compared to fewer than 26 in the other reaction sets), these subsets provide a more reliable representation of the overall error distribution. Therefore, the subset of pericyclic reactions will be further investigated below, focusing on individual outliers.

These observations can also be clearly identified in the MADs and SDs, listed in **Table** **2**. The total MAD is within the chemical accuracy of 4 kJ mol^−1^ for both reaction energies (2.76 kJ mol^−1^) and barrier heights (3.56 kJ mol^−1^). In the work done by Lampe and Neugebauer,^[^ ^48^ ^]^ it was also observed that the total MAD is lower for reaction energies (1.58 kJ mol^−1^) than for barrier heights (2.09 kJ mol^−1^), comparing pair‐selected ML calculations with a target accuracy of NormalPNO with respect to TightPNO DLPNO‐CCSD(T_0_) for the def2‐TZVP basis set.^[^ ^56^ ^]^ The broad distribution of errors due to individual outliers (mainly from the pericyclic reactions) can be recognized by high SDs of 13.17 kJ mol^−1^ for reaction energies and 13.76 kJ mol^−1^ for barrier heights.

**Table 2:** MAD, MAX, and SD in kJ mol^−1^ for reaction energies and barrier heights of ML DLPNO‐CCSD(T_0_) calculations with $\tau_{\Delta }=10^{-3}\;E_{h}$ in comparison to NormalPNO DLPNO‐CCSD(T_0_) using the aug‐cc‐pVTZ basis set. The deviations are given for subsets of reaction types and for the total set of closed‐shell reactions from the BH9 benchmark set.

Type	Reaction energies			Barrier heights		
	MAD	MAX	SD	MAD	MAX	SD
II	4.14	147.02	16.87	4.39	148.88	16.90
V	0.80	4.89	0.92	3.81	41.20	8.91
VII	0.54	2.34	0.71	1.92	16.07	4.77
VIII	0.88	2.97	0.80	0.84	3.14	0.88
IX	0.50	3.51	0.80	0.54	3.47	0.80
Total	2.76	–	13.17	3.56	–	13.76

### 4.2 Analyzing Errors from the Triples Correction

As demonstrated in the previous section, there are a few outliers with errors larger than chemical accuracy. To further analyze the origin of such cases, we investigated six of these outliers in more detail regarding the contribution of the triples to the overall error. We would like to stress that in the majority of those cases, a threshold value of $\tau_{\Delta }=10^{-4}\;E_{h}$ leads to very close agreement with the reference calculation (see Table S2, Supporting Information). Above, we already investigated the overall error introduced by the ML approach, by comparing DLPNO‐CCSD(T_0_) results for SL and ML cases. Here, we analyze the deviations for the semi‐canonical (T_0_) and iterative (T_1_) treatment of the triples by comparing the ML‐T_0_, SL‐T_0_, ML‐T_1_, and SL‐T_1_ variants of DLPNO‐CCSD(T). Our previous work^[^ ^48^ ^]^ suggested that the iterative triples correction could potentially have a positive impact in problematic cases. We have now applied the iterative method both to the DOS ML‐pair‐selected procedure used above, and to the orbital‐map version from the work done by Lampe and Neugebauer^[^ ^48^ ^]^ which is denoted as ML2 here.

**Figure 3:**
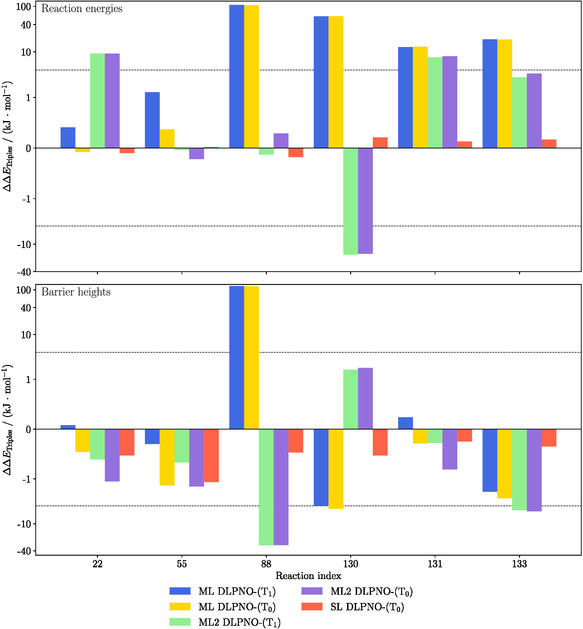
Comparison of the deviation in relative triples contributions $\Delta \Delta E_{\text{Triples}}$ for reaction energies (top) and barrier heights (bottom) for a few selected pericyclic reactions, with respect to SL DLPNO‐(T_1_ $T_{1}$). The aug‐cc‐pVTZ basis set is used. A threshold of $\tau_{\Delta }=10^{-3}\;E_{h}$ and NormalPNO settings are employed for ML; for ML2, we used the thresholds $\tau_{\text{CutPNO}}^{\Delta }=10^{-3}\;E_{h}$, $\tau_{\text{CutPairs}}^{\Delta }=10^{-5}\;E_{h}$, and $\tau_{\text{CutTriples}}^{\Delta }=10^{-5}\;E_{h}$. Note the logarithmic scale on the y‐axis.

**Figure** **3** compares the contributions of the different triples correction schemes to reaction energies and barrier heights for selected reactions from the subset of pericyclic reactions (II). For this detailed analysis, reactions with moderate system size and an absolute reaction‐energy error of more than 4 kJ mol^−1^ of the ML approach with respect to NormalPNO DLPNO‐CCSD(T_0_) were selected. The triples contributions to the SL DLPNO‐CCSD(T_0_) calculations, denoted as SL‐DLPNO‐(T_0_) here, do not show large deviations from the $T_{1}$ reference results for any of these reactions. For the ML variants, the reaction energy and barrier height errors for reactions 22 and 55 are below the 4 kJ mol^−1^ limit. For reactions with the index 88, 130, 131, and 133, however, the errors in the reaction energies are much larger, reaching more than 120 kJ mol^−1^ in one case. For the barriers, most of the errors are below or very close to the 4 kJ mol^−1^ limit. This is the case for both, ML DLPNO‐CCSD(T_1_) and ML DLPNO‐CCSD(T_0_). For the ML2 variant, some reaction energies (55, 88, 133) and some barrier heights (22, 55, 130, 133, 133) are below or close to the 4 kJ mol^−1^ threshold. For the other systems, ML2 also shows larger deviations of up to 30 kJ mol^−1^. Figure 3 shows that for most reaction energies and some of the barriers a large error originates from the triples correction. This can be observed both for the DLPNO‐(T_0_) and DLPNO‐(T_1_) correction. In these problematic cases, the origin of the large error is therefore not a consequence of the SC triples approximation. Instead, the triples‐selection procedure apparently misses some important contributions here.

To further investigate this issue, we performed additional tests using the ML2 approach, which allows a more straightforward control of the prescreening procedure in terms of the three ML thresholds $\tau_{\text{CutPNO}}^{\Delta }$, $\tau_{\text{CutPairs}}^{\Delta }$, and $\tau_{\text{CutTriples}}^{\Delta }$. The precise meanings of these cutoffs are described in the work done by Lampe and Neugebauer;^[^ ^48^ ^]^ in a nutshell, these are thresholds controlling the assignment of different sets of DLPNO thresholds $\tau_{\text{CutPNO}}$, $\tau_{\text{CutPairs}}$, and $\tau_{\text{CutTriples}}$ to orbital pairs (and triples). For infinitely sharp (i.e., zero) $\tau^{\Delta }$ thresholds, all orbital pairs and triples are treated with the most accurate DLPNO level selected (i.e., with the tightest set of DLPNO thresholds), whereas, for extremely loose $\tau^{\Delta }$ thresholds, the calculation would correspond to SL run with the most loose set of DLPNO thresholds.

As a test system, we choose reaction number 130 from the set of pericyclic reactions (set II), which contains 35 atoms, and which showed a rather large triples error of about 16.55 kJ mol^−1^ for the reaction energy in the ML2 setup (cf. Figure 3). This system was calculated with an aug‐cc‐pVTZ basis and NormalPNO settings. As a first test, we confirmed that the triples excluded by the prescreening procedure do have a negligible effect on the reaction energy and barrier. This can be seen from Figure S6, Supporting Information. While the cumulative effect of the excluded triples amounts to roughly 13 kJ mol^−1^ compared to the SL calculation, this energy offset is constant within about 0.4 kJ mol^−1^ for the three structures considered (reactant, transition state, and product). Hence, the triples excluded by the prescreening procedure do not lead to significant inaccuracies in relative energies. Subsequently, we tested the influence of the three ML thresholds on the triples included in the ML setup independently. At first, only the threshold $\tau_{\text{CutTriples}}^{\Delta }$ (compare, Figure S8, Supporting Information) was used, while the other thresholds were set to 0. As expected, this threshold exhibits no significant impact on the overall error, because 1) the discarded triples do not show a significant effect on relative energies (see above), and 2) the remaining triples contributions are treated in the same way as in a SL calculation. We therefore retain the triples threshold in all subsequent computations.

**Figure 4:**
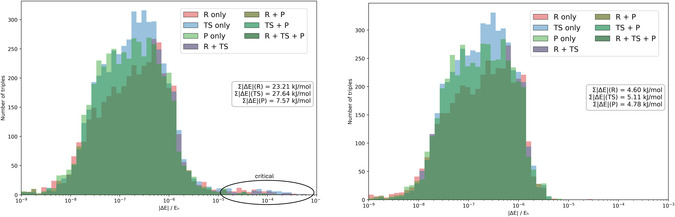
Difference of triples energy contributions for included triples between ML2 and SL DLPNO‐CCSD(T_0_) calculations for the reactant R (red), transition state TS (blue), product (green), and different color combinations (R+TS, TS+P, R+P, and R+TS+P) of the pericyclic with the reaction index 130. The energy is given in hartree atomic units ($E_{h}$) and the scale is logarithmic. The aug‐cc‐pVTZ basis set and NormalPNO settings are used. Different cutoffs for the ML2 calculations are used: i) all standard cutoffs (left) and ii) only $\tau_{\text{CutPNO}}^{\Delta }$ and $\tau_{\text{CutTriples}}^{\Delta }$. The sum of all triples contributions is listed on the right for R, TS, and P in kJ mol^−1^.

In **Figure** **4**, we compare the deviations of the triples contributions from ML2 w.r.t. SL when applying all three ML cutoffs (left) and when using only $\tau_{\text{CutPNO}}^{\Delta }$ in addition to $\tau_{\text{CutTriples}}^{\Delta }$ (right). In the former case, it can clearly be recognized that several triples exhibit errors between $10^{-5}$ and $10^{-3}$ for reactant or transition‐state structure. But these errors are not matched by corresponding errors in the product structure, and also differ between reactants and transition state. This means that error cancelation in relative energies will not work well, as can be seen from the cumulative errors listed in the inset of Figure 4 (left): the deviations to SL are significantly higher for reactants and transition state than for the products, explaining the major part of the large error in the reaction energy for this example. Further information is provided in Table S11, Supporting Information, where the 50 triples contributions with the largest errors are shown in decreasing order. This list is heavily dominated by triples from reactants and transition state, while only a few contributions from the product structure appear.

By contrast, when omitting the threshold $\tau_{\text{CutPairs}}^{\Delta }$ (equivalent to setting $\tau_{\text{CutPairs}}^{\Delta }=0$; see Figure 4, right), no problematic triples with errors in this range appear. This leads to much smaller cumulative deviations from the SL results, which, more importantly, are almost constant between the different structures (between 4.60 and 5.11 kJ mol^−1^). As a cross‐check, we also verified that omitting $\tau_{\text{CutPNO}}^{\Delta }$, and hence, only using $\tau_{\text{CutPairs}}^{\Delta }$ in addition to $\tau_{\text{CutTriples}}^{\Delta }$ does not lead to a significant improvement (see Figure S7, Supporting Information). Hence, the threshold $\tau_{\text{CutPairs}}^{\Delta }$ is identified as the critical parameter for this triples‐related error. This can be understood through its indirect effect on the triples correction: A larger (less tight) value for $\tau_{\text{CutPairs}}^{\Delta }$ implies that more orbital pairs are treated with less accurate DLPNO settings, which will typically increase the number of orbital pairs which are excluded from the pair amplitude equations (and just treated by SC MP2). In turn, the number of strong pairs will typically decrease. Since the pair amplitudes enter the perturbative triples correction, this has an indirect effect on the triples‐related error.

**Figure 5:**
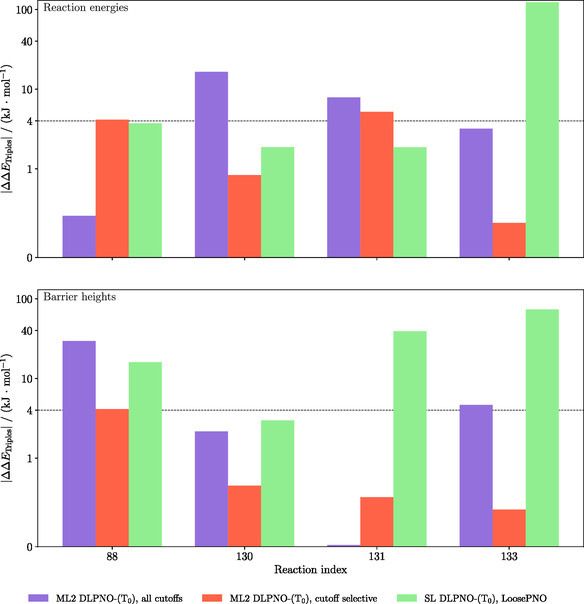
Difference DLPNO‐(T_0_) contributions for reaction energies (top) and barrier heigths (bottom) for selective systems from the pericyclic reaction set (88, 130, 131, 133). The calculations were performed for NormalPNO ML2 DLPNO‐(T_0_) with all cutoffs as before, for NormalPNO ML2 DLPNO‐(T_0_) with selective thresholds (only $\tau_{\text{CutPNO}}^{\Delta }$ and $\tau_{\text{CutTriples}}^{\Delta }$) and LoosePNO SL DLPNO‐(T_0_) with aug‐cc‐pVTZ as basis set and NormalPNO SL DLPNO‐(T_0_) as reference.

To analyze whether this effect of $\tau_{\text{CutPairs}}^{\Delta }$ is a more general phenomenon among the problematic cases identified in our benchmark, we also reinvestigated the errors arising from the triples contributions for three additional problematic systems (cf. Figure 3) from the set of pericyclic reactions (indices: 88, 131, 133). The results for all four test systems investigated here are shown in **Figure** **5**, where we compare ML2 calculations employing all cutoffs to those omitting $\tau_{\text{CutPairs}}^{\Delta }$. It can be seen that all relative‐energy errors for the ML2 calculations with selective thresholds (only $\tau_{\text{CutPNO}}^{\Delta }$ and $\tau_{\text{CutTriples}}^{\Delta }$; red bars in Figure 5) are within an acceptable range of errors, either within or at least very close to the desired chemical accuracy (4 kJ mol^−1^). For two of the cases (reaction energy for system 88 and reaction barrier for system 131), omitting $\tau_{\text{CutPairs}}^{\Delta }$ leads to a slight decrease in accuracy, which obviously must be due to accidental error cancelation taking place in the calculations with $\tau_{\text{CutPairs}}^{\Delta }$. In that figure, we also show results of LoosePNO SL DLPNO‐(T_0_), which demonstrate that ML2 with selective cutoffs avoids the cases with dramatic failures observed with LoosePNO, and offers better (or at least comparable) accuracy also in the other cases.

To conclude from these tests, $\tau_{\text{CutPairs}}^{\Delta }$ appears to be the most critical parameter, triggering the sporadic occurrence of problematic cases in our ML setup. To resolve these issues, this parameter can simply be chosen more tight (or even be set to zero). The main benefit in these calculations, which is the screening of the triples contributions, is retained in this case, since this is essentially controlled by the parameter $\tau_{\text{CutTriples}}^{\Delta }$. The corresponding solution in the ML variant, which is the main subject of this study, is to choose a tighter ML parameter $\tau_{\Delta }$, but this will typically also increase the number of triples retained.

### 4.3 Comparison of Computational Timings

The BH9 test set includes systems with a maximum size of 71 atoms, which is certainly too small to demonstrate and fully exploit the potential computational savings offered by the ML DLPNO‐CC strategy, and while the main purpose of this work is benchmarking the accuracy, which necessarily puts limitations on the system sizes that can be studied with the reference method, we compare the computational timings of the ML and reference calculations in the following. **Table** **3** presents the timings for all proton transfer reactions (VII) and the largest systems, in terms of the number of atoms, of the remaining reaction sets. Furthermore, the influence of varying the pair selection threshold $\tau_{\Delta }$ is investigated for the largest system of the pericyclic reactions (II).

**Table 3:** Comparison of time taken to calculate correlation energy differences for proton transfer reactions (VII) and for the largest reaction each of the other subsets of closed‐shell reactions from the BH9 set with NormalPNO SL DLPNO‐CCSD(T_0_) ($t_{\text{ref}}$) and the employed pair‐selected ML approach ($t_{\text{multi}}$) with the aug‐cc‐pVTZ basis set. Time is given in minutes for different reaction types with various system sizes in terms of the number of atoms. The reaction numbers (index) are adopted from Ref. [51], the ML calculations were performed with the threshold $\tau_{\Delta }=1\cdot 10^{-3}\;E_{h}$. Parenthesis indicate a modified threshold of$\tau_{\Delta }=1\cdot 10^{-4}\;E_{h}$.

Type	Index	Size	$t_{\text{ref}}$	$t_{\text{multi}}$	*t*_multi_/*t*_ref_
VIIa)	1	12	29	23	0.79
	2	15	88	66	0.75
	3	16	112	85	0.76
	4	20	25	20	0.80
	5	24	135	83	0.61
	6	28	535	403	0.75
	7	31	749	557	0.74
	8	47	664	357	0.54
	9	15	36	29	0.81
	10	25	224	127	0.57
IIb)	7	63	683	435 (626)	0.64 (0.92)
Vb)	42	71	827	693	0.84
VIIb)	8	47	195	125	0.64
VIIIb)	15	54	317	252	0.79
IXb)	26	50	186	128	0.69

a) Intel(R) Xeon(R) Silver 4216 CPU @ 2.10 GHz (32 cores).

b) Dual AMD EPYC 7643 48‐Core Processor (96 cores).

As seen in Table 3, the ML approach consistently outperforms the SL calculation in terms of computational efficiency. A systematic trend between the relative timings $t_{\text{multi}}/t_{\text{ref}}$ and the number of atoms is observable comparing different proton transfer reactions (VII). For the largest system containing 47 atoms, the computational time is reduced to 54%, whereas the relative timings of reactions with 20 or less atoms range from 75% to 81%. Comparing reactions of different subsets, this trend seems less pronounced. For example, reaction 42 of the hydride transfer reactions (V) has a relative timing of 84%, although it is the largest system. However, the relative timing mainly depends on the relative number of orbital pairs, which are identified as significant. This number depends on the redistribution of electrons during the reaction and hence on the reaction type. The effect of the relative number of significant orbital pairs is quantified in the Supporting Information (Figure S2, Supporting Information). Moreover, in the lower part of the table, ML calculations with $\tau_{\Delta }=10^{-3}\;E_{h}$ are compared to those with $\tau_{\Delta }{=10}^{-4}\;E_{h}$. At the tighter threshold of $\tau_{\Delta }=10^{-4}\;E_{h}$, the computational savings are minimal, as a large part of orbital‐pair sets are categorized as significant.

In summary, these timings show that there is a gain in computing time even for these comparatively small test systems. A full motivation for using the pair‐selected ML approach, however, arises only for larger systems beyond the size of 100 atoms, as has been demonstrated in previous work.^[^ ^44^, ^48^ ^]^ A decarboxylation reaction involving a system of 130 atoms in total will be discussed below.

### 4.4 Comparison to Canonical CCSD(T)

**Figure 6:**
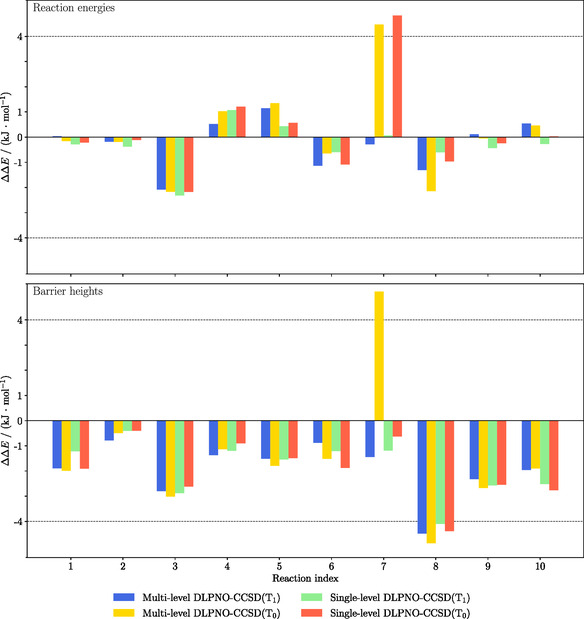
Comparison of the errors in relative triple correction energies ΔΔE for barrier heights (top) and reaction energies (bottom) with respect to CCSD(T) for the proton transfer reactions. ML DLPNO‐CCSD(T_0_), ML DLPNO‐CCSD(T_1_), and NormalPNO DLPNO‐CCSD(T_0_) and DLPNO‐CCSD(T_1_) are compared. The def2‐TZVP basis set is used. A threshold of $\tau_{\Delta }=10^{-3}\;E_{h}$ is employed.

A question that may arise is if the ML DLPNO variants do accumulate the errors from the ML treatment and the underlying SL DLPNO approximation. We address this issue by analyzing the deviations in relative energies from canonical CCSD(T) results obtained in the work done by Lampe and Neugebauer^[^ ^48^ ^]^ for the proton‐transfer reactions of the BH9 set. In **Figure** **6**, the relative reaction energies and barrier heights are shown for ML DLPNO‐CCSD(T_1_/T_0_) and SL DLPNO‐CCSD(T_1_/T_0_) in comparison to canonical CCSD(T). It can be seen that the ML variants do not introduce significant additional errors in comparison to the SL calculations for the majority of cases. This is similar to the results with ML2 from the work done by Lampe and Neugebauer.^[^ ^48^ ^]^ In some cases, for example, reactions index 2 and 9, the ML DLPNO‐CCSD(T_1_) shows even smaller deviations from canonical CCSD(T) than the SL DLPNO‐CCSD(T_1_) variant. For system 8, the error for all DLPNO variants is slightly larger than 4 kJ mol^−1^, pointing towards a DLPNO weakness rather than a specific ML problem. System 7 shows deviations above 4 kJ mol^−1^ for the SC (T_0_) case except for the SL barrier calculation. These somewhat larger errors disappear with the iterative triples correction (T_1_). Hence, this is an example where the DLPNO‐CCSD(T_0_) method shows deviations to canonical CCSD(T), which are cured by switching to (T_1_) instead. In conclusion, it can be clearly be seen that the errors in the ML treatment are dominated by the DLPNO errors, while additional ML effects are very mild and may actually lead to error cancelation.

### 4.5 Performance and Timings for a Decarboxylation Reaction

In this Section, we report errors and timings for a decarboxylation reaction^[^ ^50^ ^]^ comprising more than 130 atoms. As already noted, the application of the ML DLPNO approach to large molecular systems should provide substantially greater benefits compared to smaller benchmarks. Consequently, this system is expected to demonstrate a stronger impact than the BH9 benchmark set, thereby underscoring both the efficiency and the scalability of the method to challenging real‐life.

**Figure 7:**
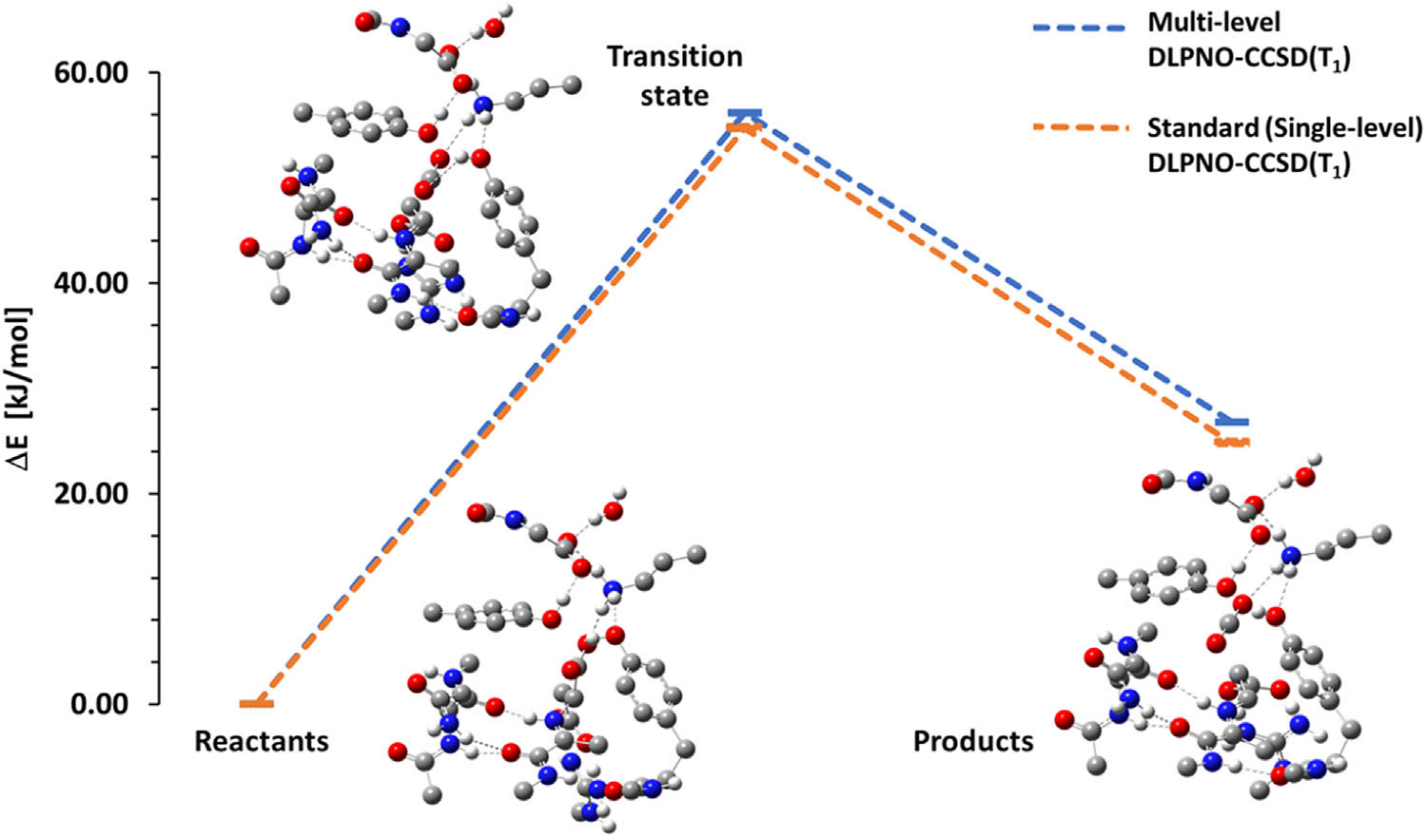
Relative energies for the decarboxylation reaction (135 atoms). The pair‐selected ML DLPNO‐CCSD(T_1_) is compared with SL DLPNO‐CCSD(T_1_) with def2‐TZVP basis set and NormalPNO settings.

**Figure** **7** and **Table** **4** provide a comprehensive overview of both accuracy and efficiency. Figure 7 summarizes the deviation for ML and SL DLPNO for barrier heights (1.46 kJ mol^−1^) and reaction energies (1.97 kJ mol^−1^). Table 4 reports the computational timings, including the total wall time as well as the contributions from individual components of the calculation. This separation allows for a direct assessment of the trade‐off between accuracy and computational cost, highlighting which steps dominate the overall runtime. It can clearly be seen that the time‐dominating step, the iterative triples correction, is reduced to ≈42% for the ML case in comparison to SL DLPNO, which represents a substantial improvement. Another aspect is that the relative timing for the DLPNO‐(T_1_) part in comparison to the whole procedure [DLPNO‐CCSD(T_1_)] is decreased from 64% (SL) to 46% (ML). This reduction is particularly promising, as for even larger molecular systems this component of the calculation typically becomes increasingly dominant. Consequently, one can expect that the overall time benefit of the present approach will improve systematically with system size, thereby enhancing its applicability to chemically relevant large‐scale problems.

**Table 4:** Timings for the decarboxylation reaction (135 atoms). ML DLPNO‐CCSD(T_1_) is compared to SL DLPNO‐CCSD(T_1_) with def2‐TZVP as basis set and NormalPNO settings. Also listed are the individual contributions of the DLPNO‐(T_0_) and DLPNO‐(T_1_) steps to the total timings.

Timings			
	$t_{\text{multi}}/t_{\text{ref}}$	$t_{\text{multi}}$ [min]	*t*_ref_ [min]
DLPNO‐CCSD(T_1_)	0.58	1155.3	1976.6
DLPNO‐(T_0_)	0.53	188.6	357.2
DLPNO‐(T_1_)	0.42	533.7	1272.9

## 5 Conclusion

In this work, the pair‐selected ML DLPNO‐CCSD(T_0_) approach from the work done by Bensberg and Neugebauer^[^ ^44^ ^]^ has been successfully tested and benchmarked for closed‐shell reactions from the comprehensive BH9 data set. In this ML approach, orbital pairs are categorized according to their SC‐MP2 pair correlation energy change and are further treated with two levels of accuracy settings, that is, NormalPNO and LoosePNO. The contributions are scrutinized employing the selection threshold $\tau_{\Delta }=10^{-3}\;E_{h}$, and rarely $\tau_{\Delta }=10^{-4}\;E_{h}$, which is the only controlling parameter in the partitioning. This specific characteristic of a direct dependence on a single parameter makes this method robust and applicable to variety of chemical reactions as demonstrated in this work. The selection threshold $\tau_{\Delta }=10^{-3}\;E_{h}$, suggested in previous studies, works effectively for the majority of tested reactions. This means that the accuracy is almost consistently within the chemical accuracy of 4 kJ mol^−1^ alongside a significant time benefit with respect to NormalPNO DLPNO‐CCSD(T_0_). However, only a tighter threshold ($\tau_{\Delta }=10^{-4}\;E_{h}$) provided reaction energies and barrier heights within an accuracy of 4 kJ mol^−1^ for some exceptional cases. As the time benefit of such a tight threshold is minor, it is of interest to analyze the origin of the errors for these outliers. Similar to previous studies with a modified pair selection scheme,^[^ ^48^ ^]^ the triples corrections contribute the most to such high errors in relative energies. As previously suggested, the SC approximation for the triples is a possible error source. For this reason, we have employed the pair‐selected ML DLPNO‐CCSD(T_1_) approach and compared the SC triples correction with the iterative triples correction. However, for the threshold $\tau_{\Delta }=10^{-3}\;E_{h}$, the triples errors for the exceptional cases found here are almost identical for SC and iterative triples for both ML methods (DOS‐based ML and ML2). One possible explanation is that important triples contributions are discarded by the employed orbital‐pair based selection, and hence, the selection of triples provides potential for future improvements. A possible approach is to modify the conditions for an orbital triple to enter the calculation, for example, based on SC triples from a more inexpensive calculation. In combination with the improved pair selection approach,^[^ ^48^ ^]^ then, a highly robust and black‐box‐like ML method for calculating relative energies becomes feasible.

One initial motivation for our study was the observation of several problematic cases in connection with the ML2 method in the work done by Lampe and Neugebauer,^[^ ^48^ ^]^ which was somewhat surprising as we considered ML2 as more robust and accurate than the DOS‐based ML. Here, however, it was demonstrated that these problems also occur in the original ML method. This triggered a closer investigation of possible error sources within the ML treatment, which can straightforwardly be done within the ML2 method. In particular, we have identified the $\tau_{\text{CutPairs}}^{\Delta }$ ML threshold as a critical parameter for the problematic systems identified here, pointing to the fact that the observed problems are due to inaccurate pair amplitudes in the calculation of the triples correction. Adjusting this parameter in the ML2 approach, including the possibility of entirely omitting this threshold, offers a promising way of reaching chemical accuracy also in these problematic cases, while still retaining the efficiency benefits through the prescreening of triples. The latter will dominate the computational cost in larger systems. Within the original ML method, a straightforward solution for the problematic cases consists of adjusting the threshold $\tau_{\Delta }$ to a tighter value ($10^{-4}\;E_{h}$) as discussed above, but this usually significantly reduces the efficiency gains.

In conclusion, we argue that using the ML2 variant is preferable in general, since its pair‐selection procedure is more robust, offering a clear framework for systematically adjusting and mitigating these errors in future applications. Nevertheless, also the ML variant offers highly accurate relative energies in most of the test systems. The accuracy and application potential of the method are illustrated by the comparison to CCSD(T) reference calculations and by the application to the model‐system from the decarboxylation reaction. The relative errors between ML and SL DLPNO seems very promising in these cases. Especially important is that the errors from the ML calculations do not simply add up with the SL DLPNO error and can still reduce the computational cost to about 42% of the reference for the decarboxylation reaction investigated here.

## Supporting Information

The Supporting Information contains details about the number of selected pairs and their relation to computational timings and about the effect of the pair selection threshold on the number of selected triples, tables with barrier heights and reaction energies for all reactions considered, including tables for the problematic cases comparing SL, ML, and ML2 as well as T_0_ and T_1_ approaches for the triples correction, a table with the DLPNO thresholds employed in the ML2 calculations, an example input and a representation of the workflow, figures showing the errors as a function of the system size, a table with details regarding the errors in the triples contributions for reaction 130, further graphs illustrating the effect of different thresholds on the triples errors, and a table with cumulative triples contribution for the four reactions selected for detailed error analysis.

## Conflict of Interest

The authors declare no conflict of interest.

## Supporting information

Supplementary Material

## Data Availability

The data that support the findings of this study are available in the supplementary material of this article.
