# Supplementary material for: Accuracy and Limitations of the Pair‐Selected Multilevel Approach for DLPNO Coupled Cluster: Extensive Benchmark for Closed‐Shell Organic Reactions
Source: Chemphyschem. 2025 Nov 18;27(1):e202500246. doi: 10.1002/cphc.202500246 (PMC12810467; doi:10.1002/cphc.202500246)
Supplement: Supplementary file 1 — Supplementary Material [file CPHC-27-e202500246-s001.pdf]

Supporting information: Accuracy and Limitations of the Pair-Selected Multi-Level Approach for DLPNO Coupled Cluster: Extensive Benchmark for Closed-Shell Organic Reactions

Nalini Gurav[†], Nadim Ramez[†], Lukas Lampe, and Johannes Neugebauer*

Theoretische Organische Chemie, Organisch-Chemisches Institut and Center for Multiscale Theory and Computation, Universität Münster, Corrensstraße 36, 48149 Münster, Germany

E-mail: j.neugebauer@uni-muenster.de

[†]Both authors contributed equally to this work.

1 Correlation between timings and pair selection

Table S1: Comparison of the number of orbitals pairs and the time taken to calculate correlation energy differences for proton transfer reactions (VII) and for the largest reaction each of the other subsets of closed-shell reactions from the BH9 set with NormalPNO DLPNO-CCSD(T_0) (t_{ref}) and the employed pair-selected multi-level approach (t_{multi}) with the aug-cc-pVTZ basis set. Time is given in minutes for different reaction types with various system sizes in terms of the number of atoms. The reaction indices are adopted from Ref. 1. The multi-level calculations were performed with the threshold $\tau_\Delta = 1 \cdot 10^{-3}\ E_{\mathrm{h}}$. Number in parentheses correspont to a modified threshold of $\tau_\Delta = 1 \cdot 10^{-4}\ E_{\mathrm{h}}$. The total number of orbital pairs is denoted by n_{pairs} and the number of significant orbital pairs with regard to τ_Δ is denoted by $n_{\mathrm{pairs}}^{e>\tau_\Delta}$.

Type	Index	Size	t_{ref}	t_{multi}	$t_{\mathrm{multi}}/t_{\mathrm{ref}}$	n_{pairs}	$n_{\mathrm{pairs}}^{e>\tau_\Delta}$	$n_{\mathrm{pairs}}^{e>\tau_\Delta}/n_{\mathrm{pairs}}$
VII[a]	1	12	29	23	0.79	178	70	0.39
	2	15	88	66	0.75	361	75	0.21
	3	16	112	85	0.76	417	98	0.24
	4	20	25	20	0.80	360	200	0.56
	5	24	135	83	0.61	446	37	0.08
	6	28	535	403	0.75	1398	307	0.22
	7	31	749	557	0.74	1561	500	0.32
	8	47	664	357	0.54	2511	300	0.12
	9	15	36	29	0.81	261	123	0.47
	10	25	224	127	0.57	835	170	0.20
II[b]	7	63	683	435 (626)	0.64 (0.92)	3269	278 (953)	0.09 (0.29)
V[b]	42	71	827	693	0.84	4039	652	0.16
VII[b]	8	47	195	125	0.64	2511	300	0.12
VIII[b]	15	54	317	252	0.79	3431	756	0.22
IX[b]	26	50	186	128	0.69	2803	351	0.13

[a]Intel(R) Xeon(R) Silver 4216 CPU @ 2.10GHz (32 cores).

[b]Dual AMD EPYC 7643 48-Core Processor (96 cores).

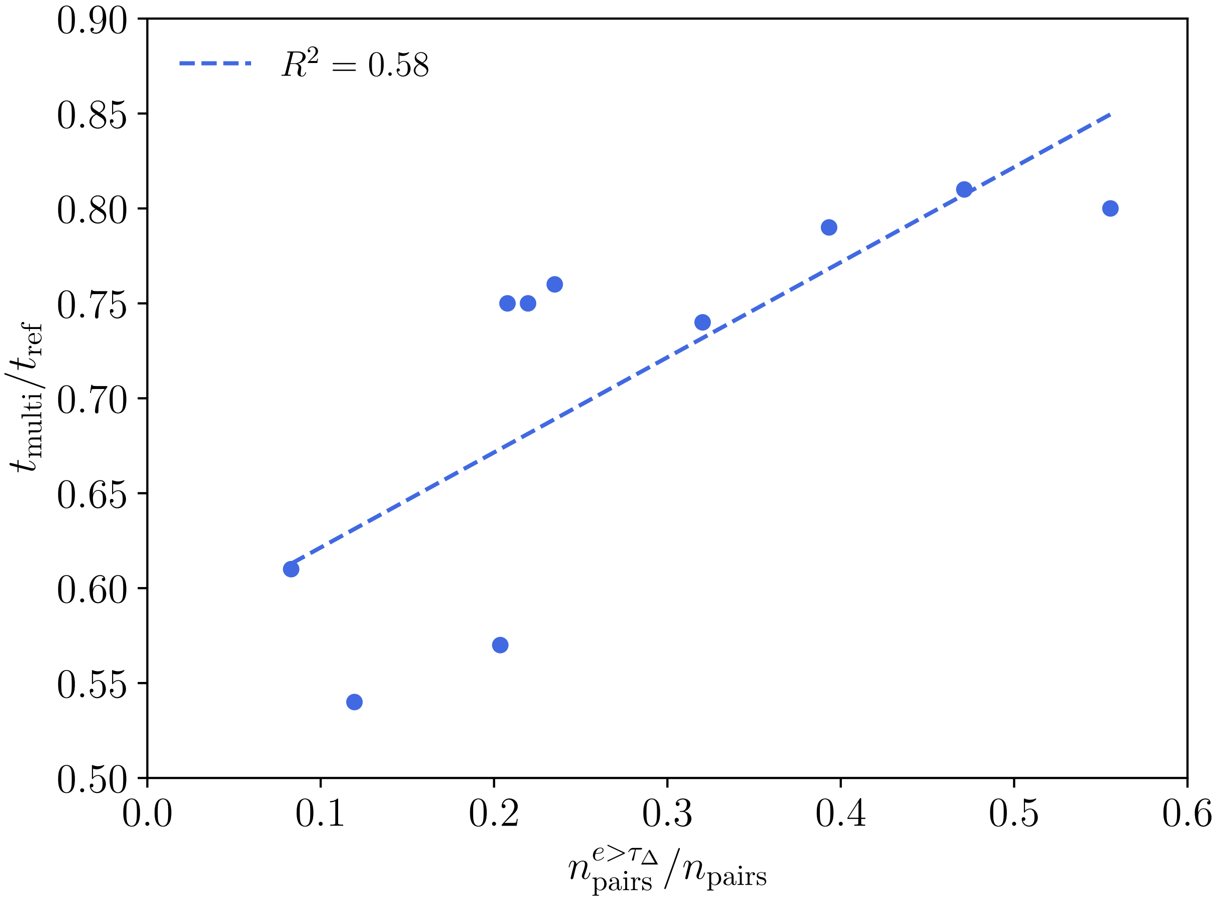

Figure S1: Relative timings $t_{\mathrm{multi}}/t_{\mathrm{ref}}$ plotted against the ratio between selected significant pairs and the total number of pairs $n_{\mathrm{pairs}}^{e>\tau_\Delta}/n_{\mathrm{pairs}}$ for the pair-selected multi-level approach with $\tau_\Delta = 1 \cdot 10^{-3}$ E_h with respect to NormalPNO DLPNO-CCSD(T_0) calculated with the aug-cc-pVTZ basis set. All proton transfer reactions (VII) are shown.

2 Effect of the pair selection threshold on the number of pairs and number of triples

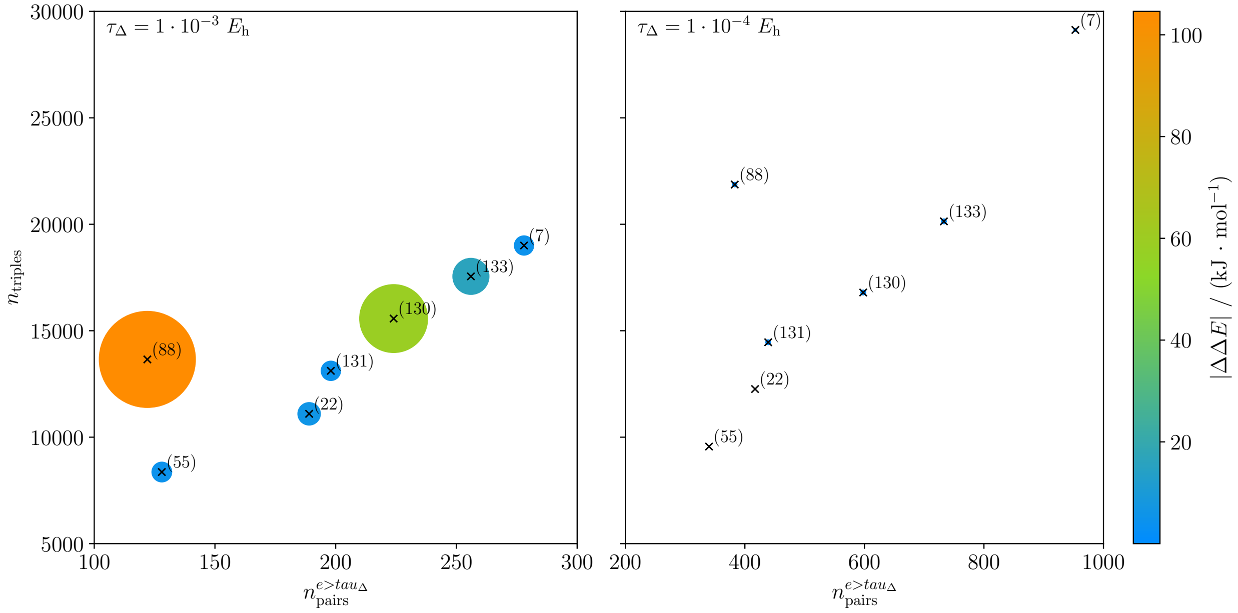

Figure S2: Total number of triples n_triples (reactant, transition state, and product combined) is plotted against the number of significant pairs $n_\mathrm{pairs}^{e>\tau_\Delta}$ for a few selected pericyclic reactions (reaction indices are given in parenthesis) using pair-selected multi-level DLPNO-CCSD(T_0) with two different pair selection thresholds τ_Δ. The colormap and circle size both indicate the corresponding absolute reaction-energy error. The aug-cc-pVTZ basis set is used.

3 Barrier heights and reaction energies

Table S2: Barrier heights and reaction energies in kJ/mol of the single-level (SL) DLPNO-CCSD(T_0) reference and pair-selected multi-level (ML) DLPNO-CCSD(T_0), calculated with the aug-cc-pVTZ basis set, for pericyclic reactions (II) from the BH9 benchmark set. The multi-level calculations were performed with the threshold $\tau_\Delta = 1 \cdot 10^{-3}\ E_\mathrm{h}$. Numbers in parentheses correspond to modified threshold of $\tau_\Delta = 1 \cdot 10^{-4}\ E_\mathrm{h}$. The reaction indices are adopted from Ref. 1.

	Reaction energies		Barrier heights	
Reaction index	SL	ML	SL	ML
1	−166.69	−165.64	10.38	11.00
2	−161.75	−161.63	12.47	12.30
3	−168.45	−167.57	22.76	23.81
4	−170.12	−169.54	16.11	16.78
5	−270.91	−272.55	110.50	109.62
6	−138.07	−142.00	113.55	110.62
7	39.08	45.86(39.33)	−88.95	−94.52(−88.91)
8	−106.36	−105.98	44.85	44.60
9	−136.69	−134.35	60.71	62.89
10	−143.80	−143.09	66.99	69.25
11	−109.12	−108.99	80.71	80.79
12	−95.94	−96.27	89.45	88.28
13	−158.24	−157.32	90.29	97.32
14	−169.45	−170.25	76.86	78.91
15	−160.96	−164.10	86.48	85.35
16	−128.03	−129.87	89.87	89.41
17	−185.02	−192.84	57.24	54.98
18	−214.60	−215.02	76.07	73.05
19	−160.08	−161.25	82.42	81.17

20	−162.63	−152.55	72.89	85.35
21	−205.77	−204.47	48.58	48.83
22	35.23	35.19(35.23)	−244.64	−250.87(−244.64)
23	−136.06	−133.97	55.65	94.98
24	−199.16	−199.87	104.14	104.27
25	−132.01	−134.22	90.25	89.45
26	−87.74	−87.49	106.48	106.73
27	−91.50	−93.22	112.84	110.62
28	−69.62	−69.04	86.32	86.11
29	−9.20	−8.74	−153.72	−151.54
30	−208.78	−208.61	21.97	20.42
31	−107.40	−106.40	43.64	45.44
32	−209.95	−209.83	14.69	15.19
33	−173.64	−173.18	42.59	43.01
34	−55.40	−55.52	73.55	74.14
35	−57.57	−57.70	67.03	67.49
36	−79.08	−81.84	61.97	61.21
37	−78.49	−80.29	68.32	67.49
38	−50.96	−48.07	95.44	95.48
39	−11.92	−11.63	152.17	152.55
40	−76.36	−76.48	41.30	41.30
41	−34.06	−33.26	98.24	98.16
42	−179.58	−179.37	17.78	17.91
43	−151.08	−150.79	48.83	49.08
44	−166.98	−166.77	53.05	53.22
45	−188.41	−187.95	57.20	57.74
46	−112.42	−111.96	96.32	96.99

47	−485.80	−486.56	−853.70	−854.00
48	−10.75	−10.33	−368.74	−368.65
49	−155.39	−154.72	34.31	34.89
50	−69.50	−73.39	73.43	21.42
51	−17.03	−19.50	−108.91	−111.59
52	−107.74	−107.70	38.95	39.37
53	−119.79	−120.37	25.69	26.23
54	−7.82	−7.24	−115.85	−116.06
55	44.81	46.86(45.06)	−168.11	−163.30(−168.11)
56	−335.51	−335.31	42.84	43.68
57	−324.68	−323.84	23.51	24.23
58	−218.36	−218.03	58.91	59.45
59	−219.07	−218.28	57.78	58.49
60	−343.26	−340.62	13.35	15.15
61	−6.19	−6.40	−241.96	−242.42
62	−233.59	−233.68	6.74	7.15
63	−97.15	−97.07	72.68	72.93
64	−188.07	−188.15	782.12	782.20
65	−201.00	−201.33	33.68	33.01
66	−208.57	−208.70	24.31	26.40
67	−133.01	−180.79	7.70	12.80
68	−127.24	−124.89	15.98	19.33
69	−115.44	−131.13	39.92	40.58
70	−47.57	−44.22	122.84	105.44
71	−31.38	−34.48	127.90	123.55
72	−93.64	−93.35	112.21	124.22
73	−160.04	−159.95	29.87	29.96

74	5.56	6.61	115.81	117.53
75	−147.19	−146.69	26.86	26.61
76	−71.13	−70.92	84.43	87.15
77	−178.99	−178.74	14.43	14.23
78	−81.67	65.19	76.48	225.35
79	−7.57	−5.82	−183.51	−180.41
80	−57.20	−57.03	113.51	112.38
81	−146.44	−146.77	33.01	32.89
82	−128.49	−127.70	30.59	32.80
83	−115.94	−112.30	69.12	71.59
84	−101.55	−101.92	106.98	105.73
85	−89.70	−90.71	122.76	122.26
86	−99.87	−101.04	101.67	88.32
87	−96.65	−98.83	118.57	119.37
88	94.43	−24.85(95.98)	−112.30	−229.87(−112.51)
89	−98.62	−102.63	118.28	96.27
90	−102.47	−100.42	113.18	114.47
91	−93.43	−92.76	127.90	131.00
92	−119.33	−116.90	97.28	99.70
93	−104.06	−105.94	118.28	119.54
94	−115.94	−115.48	101.34	96.23
95	−112.55	−106.06	117.24	122.63
96	−19.41	−19.08	42.97	42.09
97	85.35	86.36	176.61	176.86
98	−52.22	−51.55	135.56	136.61
99	−43.30	−42.34	180.71	181.46
100	64.39	63.81	148.66	148.24

101	−20.04	−20.25	168.78	169.54
102	13.22	13.31	162.05	162.13
103	0.46	0.84	148.57	149.16
104	−111.46	−111.75	52.80	52.84
105	27.70	28.12	200.16	200.50
106	−101.84	−100.79	19.08	19.66
107	−127.61	−127.03	19.08	19.75
108	−116.52	−116.44	19.08	19.33
109	−18.41	−18.58	178.61	178.91
110	−57.91	−57.24	178.61	179.95
111	−58.16	−58.03	152.09	152.30
112	−100.12	−99.62	21.97	22.55
113	−122.30	−121.59	22.01	22.38
114	−128.70	−128.41	21.97	22.68
115	−100.08	−99.58	21.13	21.59
116	−123.14	−122.51	21.13	24.23
117	−137.74	−137.44	21.05	21.71
118	−42.09	−37.45	125.65	129.24
119	−127.61	−126.69	37.57	53.68
120	−117.74	−112.21	54.56	59.91
121	−163.59	−161.67	51.97	58.37
122	−83.09	−82.63	70.63	68.53
123	−103.76	−106.82	70.63	72.01
124	−102.93	−102.05	84.77	84.39
125	−108.66	−109.08	84.77	84.22
126	−21.63	−22.80	78.83	80.75
127	−40.46	−43.85	53.56	59.29

128	−39.79	−38.49	129.08	130.16
129	−29.00	−28.83	135.14	135.73
130	140.16	138.83(140.29)	−38.95	−97.45(−38.70)
131	127.03	127.07(127.15)	−51.25	−55.86(−51.00)
132	−41.17	−41.38	128.32	128.49
133	133.39	137.03(133.43)	−52.97	−69.29(−52.72)
134	−75.56	−75.56	128.32	128.74
135	−72.76	−72.43	109.12	109.04
136	−68.53	−68.66	117.65	117.44
137	−68.32	−69.66	95.77	94.43
138	8.54	8.66	126.31	126.48
139	−74.27	−73.93	133.64	133.93
140	−368.48	−370.54	225.81	226.19

Table S3: Barrier heights (BH) and reaction energies (RE) in kJ/mol of the single-level (SL) DLPNO-CCSD(T_0) reference and pair-selected multi-level (ML) DLPNO-CCSD(T_0), calculated with the aug-cc-pVTZ basis set, for hydride transfer reactions (V) from the BH9 benchmark set. The multi-level calculations were performed with the threshold $\tau_\Delta = 1 \cdot 10^{-3}$ E_h. The reaction indices are adopted from Ref. 1.

	Reaction energies		Barrier heights	
Reaction index	SL	ML	SL	ML
1	−24.10	−24.14	4.85	4.69
2	−5.73	−5.82	93.26	93.22
3	−16.90	−16.82	61.97	61.84
4	−83.93	−83.35	20.54	21.71
5	−50.04	−50.25	−0.50	−0.63
6	−7.45	−5.48	41.38	42.59
7	66.11	65.77	55.77	55.27

8	−32.09	−32.01	3.51	3.93
9	−47.07	−47.20	−10.50	−10.59
10	30.17	30.50	34.02	34.85
11	69.83	70.29	22.80	23.89
12	−55.06	−54.94	10.42	9.67
13	31.59	31.92	62.13	61.84
14	−31.21	−30.59	24.64	24.10
15	−13.72	−13.14	46.48	33.72
16	−6.11	−5.98	49.62	50.12
17	35.90	36.02	24.23	25.36
18	480.49	480.87	63.30	60.29
19	−1.30	−0.17	32.01	31.17
20	−39.46	−38.79	6.28	5.94
21	−60.42	−59.96	5.77	6.36
22	35.23	35.94	64.81	67.91
23	−30.88	−30.59	37.57	37.28
24	44.89	44.39	39.08	39.29
25	−53.89	−52.59	25.82	26.07
26	−47.70	−47.03	−0.71	−41.88
27	−65.69	−64.10	−8.62	−6.57
28	−3.56	−3.68	30.75	29.50
29	23.47	24.43	40.46	41.67
30	−36.90	−35.90	7.03	7.57
31	−4.10	−3.43	45.61	21.63
32	−53.89	−53.30	8.33	6.74
33	−67.15	−66.61	−40.25	−42.76
34	−76.57	−75.35	1.42	2.93

35	−77.82	−76.99	−12.59	−12.43
36	−1.88	−5.36	−32.72	4.52
37	−127.15	−127.49	−14.35	−17.20
38	−31.51	−31.51	20.29	23.30
39	−51.25	−51.51	3.26	5.06
40	−41.09	−45.65	−74.01	−81.50
41	−12.59	−11.38	48.41	47.53
42	−36.02	−33.26	13.39	12.55

Table S4: Barrier heights (BH) and reaction energies (RE) in kJ/mol of the single-level (SL) DLPNO-CCSD(T_0) reference and pair-selected multi-level (ML) DLPNO-CCSD(T_0), calculated with the aug-cc-pVTZ basis set, for proton transfer reactions (VII) from the BH9 benchmark set. The multi-level calculations were performed with the threshold $\tau_\Delta = 1 \cdot 10^{-3}$ E_h. The reaction indices are adopted from Ref. 1.

	Reaction energies		Barrier heights	
Reaction index	SL	ML	SL	ML
1	−3.47	−3.60	156.82	156.94
2	−0.17	0.08	3.43	3.64
3	2.57	2.72	157.86	158.16
4	49.04	49.08	47.57	47.91
5	−253.89	−253.72	49.41	50.71
6	116.02	115.85	128.66	127.86
7	−124.56	−123.89	12.51	12.38
8	−3.64	−2.13	−136.69	−152.84
9	−3.47	−3.64	18.07	18.12
10	73.35	75.69	70.04	70.08

Table S5: Barrier heights (BH) and reaction energies (RE) in kJ/mol of the single-level (SL) DLPNO-CCSD(T_0) reference and pair-selected multi-level (ML) DLPNO-CCSD(T_0), calculated with the aug-cc-pVTZ basis set, for nucleophilic substitutions (VIII) from the BH9 benchmark set. The multi-level calculations were performed with the threshold $\tau_\Delta = 1 \cdot 10^{-3}$ E_h. The reaction indices are adopted from Ref. 1.

	Reaction energies		Barrier heights	
Reaction index	SL	ML	SL	ML
1	−75.23	−75.19	−13.64	−13.47
2	59.66	59.79	57.03	57.32
3	−59.12	−59.16	54.31	54.64
4	−32.68	−32.22	93.81	94.18
5	−41.34	−39.50	−24.73	−22.93
6	43.68	42.63	80.58	80.46
7	−12.93	−12.68	9.29	12.43
8	3.85	3.31	62.72	63.55
9	40.00	39.54	9.37	9.41
10	−12.97	−14.18	7.53	5.44
11	−93.93	−94.73	−17.03	−18.41
12	26.99	25.69	41.30	40.08
13	−23.10	−23.43	44.85	44.69
14	141.46	140.04	119.33	119.16
15	65.52	62.55	70.12	69.45

Table S6: Barrier heights (BH) and reaction energies (RE) in kJ/mol of the single-level (SL) DLPNO-CCSD(T_0) reference and pair-selected multi-level (ML) DLPNO-CCSD(T_0), calculated with the aug-cc-pVTZ basis set, for nucleophilic additions (IX) from the BH9 benchmark set. The multi-level calculations were performed with the threshold $\tau_\Delta = 1 \cdot 10^{-3}\ E_\text{h}$. The reaction indices are adopted from Ref. 1.

	Reaction energies		Barrier heights	
Reaction index	SL	ML	SL	ML
1	−25.52	−25.52	−5.82	−6.02
2	−45.31	−45.35	−20.67	−20.63
3	−42.47	−42.38	80.21	80.25
4	−37.15	−37.11	65.40	65.44
5	−35.23	−35.23	73.43	73.43
6	−108.37	−108.32	24.73	24.77
7	−66.78	−66.61	4.94	4.90
8	−59.54	−59.37	4.06	4.02
9	−111.96	−111.88	24.06	24.23
10	−27.20	−27.53	0.71	0.88
11	79.04	79.45	132.72	132.93
12	50.79	52.51	51.76	51.84
13	−15.27	−14.35	22.76	23.77
14	−45.77	−45.94	12.05	11.72
15	−23.26	−23.43	9.00	9.12
16	−41.76	−42.09	10.38	10.17
17	−93.30	−93.43	34.48	34.10
18	−86.40	−86.23	17.91	18.03
19	37.99	37.78	80.29	80.50
20	−43.81	−40.25	−0.50	−1.88
21	−46.82	−46.28	1.13	1.97

22	35.61	35.69	48.91	47.61
23	−27.11	−27.95	26.02	24.73
24	−64.94	−63.68	4.90	6.78
25	−18.07	−18.12	16.07	16.15
26	−1.42	−3.22	20.50	17.03

Table S7: Reaction energies in kJ/mol of the triples contributions from single-level DLPNO-(T_1) reference and multi-level (ML) DLPNO-(T_1)/DLPNO-(T_0) and multi-level2 (ML2) DLPNO-(T_1)/DLPNO-(T_0), calculated with aug-cc-pVTZ basis set, for a selection of reactions from the subset of pericyclic reactions (II) from the BH9 benchmark set. The calculations were performed with the threshold $\tau_\Delta = 1 \cdot 10^{-3}$ and NormalPNO settings. The reaction indices are adopted from Ref. 1.

Reaction Index	ML-T0	ML-T1	ML2-T0	ML2-T1	SL-T0	SL-T1
22	9.2575	8.7665	−0.0152	−0.121	9.2811	9.1787
55	10.1447	9.2024	10.7383	10.5571	10.4994	10.5151
88	−103.0561	−104.4240	2.9143	3.3396	3.3816	3.2057
130	−64.9627	−64.5610	12.1639	12.8361	−4.3898	−4.1824
131	−16.6903	−16.4409	−11.6703	−11.2476	−3.7755	−3.6468
133	−22.2537	−22.4034	−6.8631	−6.2697	−3.6652	−3.4998

Table S8: Barrier heights in kJ/mol of the triples contributions from single-level (SL) DLPNO-(T_1) reference and multi-level (ML) DLPNO-(T_1)/DLPNO-(T_0) and multi-level2 (ML2) DLPNO-(T_1)/DLPNO-(T_0), calculated with aug-cc-pVTZ basis set, for a selection of reactions from the subset of pericyclic reactions (II) from the BH9 benchmark set. The calculations were performed with the threshold $\tau_\Delta = 1 \cdot 10^{-3}$ and NormalPNO settings. The reaction indices are adopted from Ref. 1.

Reaction Index	ML-T0	ML-T1	ML2-T0	ML2-T1	SL-T0	SL-T1
22	−15.8449	−16.3884	−15.1683	−15.6941	−15.7793	−16.3044
55	−20.3686	−21.4661	−20.2925	−21.0985	−20.5892	−21.7680
88	−141.2834	−142.9900	8.0498	8.1994	−21.5606	−22.0279
130	−23.8133	−24.4723	−30.2615	−30.1040	−27.9301	−28.4578
131	−27.0453	−27.5835	−26.5307	−27.0610	−27.0847	−27.3393
133	−24.8661	−25.6380	−22.2931	−22.5557	−27.2317	−27.5783

Table S9: Reaction energies in kJ/mol for proton transfer reactions (VII) from the BH9 benchmark set. The calculations were performed for the semi-canonical and iterative DLPNO calculation, for single-level (SL) and multi-level (ML) variants, and CCSD(T). A def2-TZVP and NORMALPNO for the DLPNO settings been used calculations. The reaction indices are adopted from Ref. 1.

Index	SL DLPNO-(T_0)	ML DLPNO-(T_0)	SL DLPNO-(T_1)	ML DLPNO-(T_1)	CCSD(T)
1	−2.85	−2.89	−2.76	−3.10	−3.05
2	−1.38	−1.30	−1.09	−1.30	−1.46
3	4.31	4.31	4.48	4.23	2.13
4	44.56	44.73	44.69	45.27	45.77
5	−249.20	−249.99	−249.07	−249.78	−248.66
6	117.40	116.94	116.90	117.44	116.32
7	−132.09	−130.71	−125.35	−124.98	−125.27
8	−2.97	−1.80	−3.31	−2.64	−3.93
9	−2.80	−3.01	−2.64	−3.18	−3.05
10	74.27	73.85	74.60	73.76	74.31

Table S10: Barrier heights in kJ/mol for proton transfer reactions (VII) from the BH9 benchmark set. The calculations were performed for the semi-canonical and iterative DLPNO calculation, for single-level (SL) and multi-level (ML) variants, and CCSD(T). A def2-TZVP and NORMALPNO for the DLPNO settings been used calculations. The reaction indices are adopted from Ref. 1.

Index	SL DLPNO-(T_0)	ML DLPNO-(T_0)	SL DLPNO-(T_1)	ML DLPNO-(T_1)	CCSD(T)
1	156.15	156.23	155.44	156.15	154.22
2	1.88	1.97	1.88	2.26	1.46
3	157.40	157.78	157.65	157.57	154.77
4	44.48	44.73	44.77	44.98	43.60
5	50.54	50.84	50.58	50.54	49.04
6	128.87	128.53	128.20	127.90	127.03
7	10.88	2.05	11.42	11.72	10.25
8	-135.23	-133.34	-136.06	-134.89	-140.33

9	17.24	17.36	17.28	17.03	17.03
10	67.32	66.48	67.11	66.53	66.53

4 Example Input and Workflow

```
+system
  name react
  geometry R.xyz
  charge 0
  spin 0
  +basis
    label def2-tzvp
  -basis
-system

+system
  name trans
  geometry TS.xyz
  charge 0
  spin 0
  +basis
    label def2-tzvp
  -basis
-system

+system
  name prod
  geometry P.xyz
  charge 0
  spin 0
  +basis
```

```
    label def2-tzvp
  -basis
-system

# This task performs the correlation treatment
# with the N(T)/L(T)/HF scheme denoted in the paper.
+task doscc
  act react
  act trans
  act prod
  dosSettings spread  # Settings for the orbital map construction.
  # The orbital pairs will be sorted into 3 sets.
  # 1. pair energy change > 1e-3
  # 2. pair energy change > 0. (so everything but core electron pairs if a
      frozen core is used.)
  # 3. all remaining pairs if any.
  pairCutOff {1e-3 0.0}
 orbitalPairAnalysis true
 # Toggles some additional output for the correlation energy change and
 # activate the pair-selected CC
  +loc              # orbital localization settings
    locType ibo
    splitValenceAndCore true
  -loc
  # The settings to run a rough approximation for the pair energies.
  # We will use these pair energies to then identify the pairs for
  # which the correlation energy changes. We use semi-canonical MP2
  # here, because it is very very fast.
  +lc0
    method SC-MP2
    pnoSettings loose
    useFrozenCore true
    diisStartResidual 10
```

```
  linearScalingSigmaVector true
-lc0
+lc1
  method SC-MP2
  pnoSettings loose
  useFrozenCore true
-lc1
+lc2
  method SC-MP2
  pnoSettings loose
  useFrozenCore true
-lc2
+lc3
  method none
  pnoSettings loose
  useFrozenCore true
-lc3
# The settings for the accurate correlation treatment.
# As mentioned above, we have three sets of orbital pairs.
# Each input block below provides the settings for one of
# these sets. The index in the input block name, e.g., 0
# for PairLC0, is the index of the orbital set. (Starting from 0)
+PairLC0
  method DLPNO-CCSD(T)              # The correlation method.
  pnoSettings normal                 # The accuracy of the local-CC
     approximation.
  useFrozenCore true                 # Frozen core, yes or no.
-PairLC0
+PairLC1
  method DLPNO-CCSD(T)
  pnoSettings loose
  useFrozenCore true
-PairLC1
```

```
  +PairLC2
    method none
    pnoSettings loose
    useFrozenCore true
  -PairLC2

-task
```

Table S11: DLPNO thresholds in E_h for different levels of ML2 DLPNO-CCSD($\mathrm{T}_{0/1}$) using a target accuracy of NormalPNO with the multi-level thresholds $\tau^{\Delta}_{\mathrm{CutPNO}} = 10^{-3}E_\mathrm{h}$, $\tau^{\Delta}_{\mathrm{CutPairs}} = 10^{-5}E_\mathrm{h}$, and $\tau^{\Delta}_{\mathrm{CutTriples}} = 10^{-5}E_\mathrm{h}$.

Setting	Level 1	Level 2	Level 3
τ_{CutPNO}	$3.33 \cdot 10^{-7}$	$1 \cdot 10^{-6}$	$1 \cdot 10^{-6}$
τ_{CutPairs}	10^{-4}	$1 \cdot 10^{-4}$	$1 \cdot 10^{-3}$
$\tau_{\mathrm{CutTriples}}$	10^{-6}	10^{-6}	∞

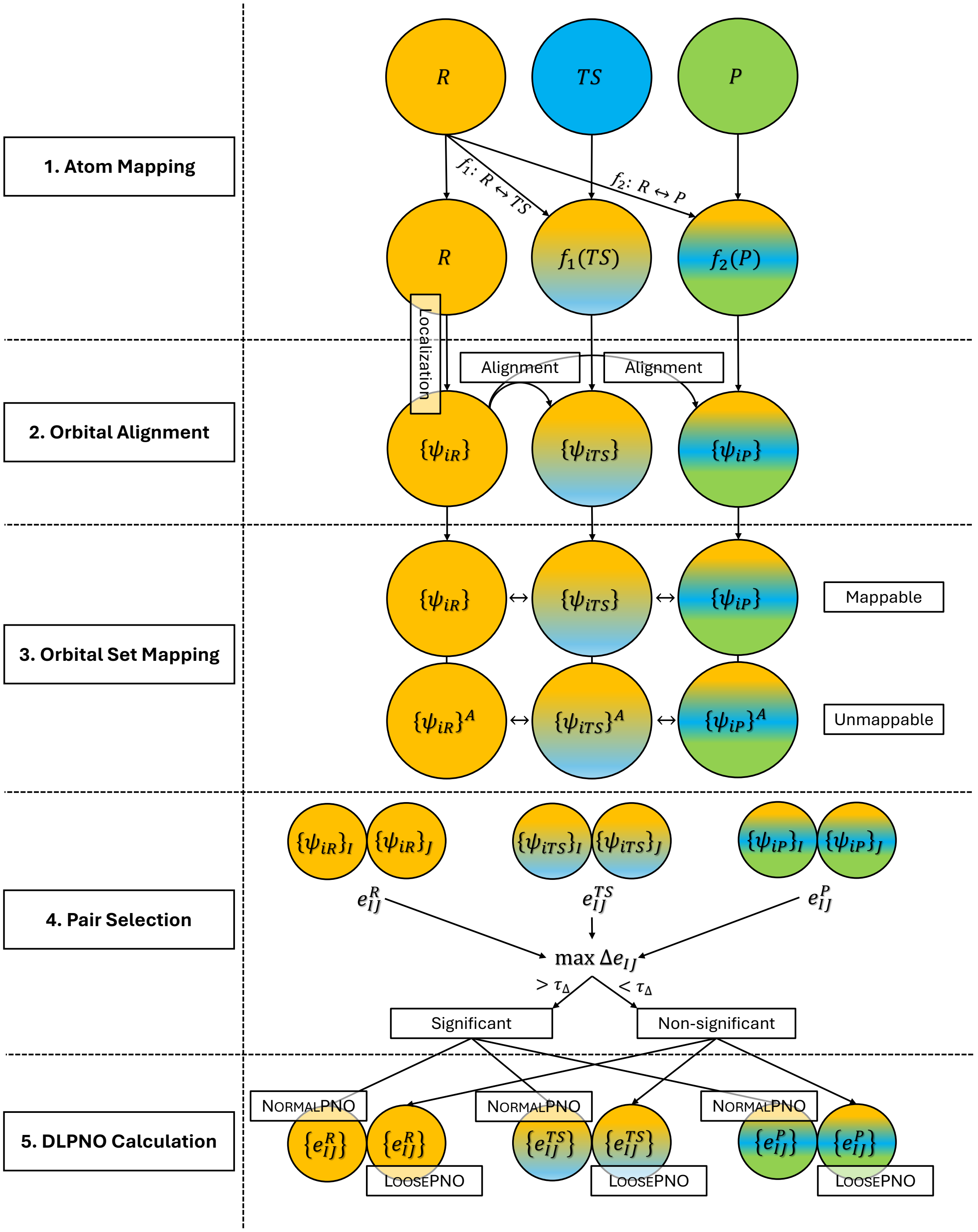

Figure S3: Schematic representation and workflow of a pair-selected multi-level calculation.

5 Error in Comparison to System Size

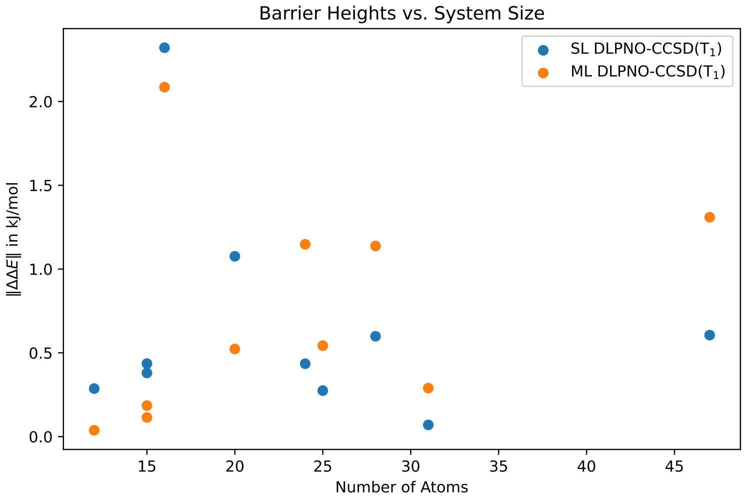

Figure S4: Absolute deviations for single-level (SL) DLPNO-CCSD(T_1) and multi-level (ML) DLPNO-CCSD(T_1) compared to CCSD(T) calculations for barrier heights plotted against the system size for the proton-transfer reactions from the BH9 benchmark set.[1] A def2-TZVP and NormalPNO settings have been used for DLPNO calculations.

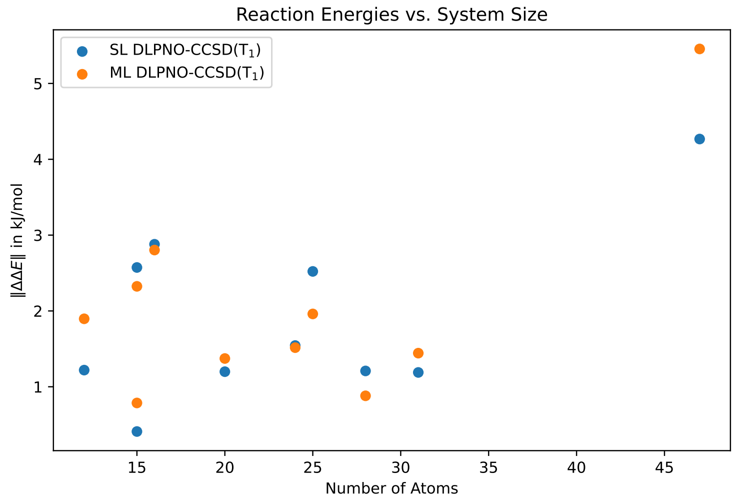

Figure S5: Absolute deviations for single-level (SL) DLPNO-CCSD(T_1) and multi-level (ML) DLPNO-CCSD(T_1) compared to CCSD(T) calculations for reaction energies plotted against the system size for the proton-transfer reactions from the BH9 benchmark set.[1] A def2-TZVP and NormalPNO settings have been used for DLPNO calculations.

6 Cutoff Controlled Error Analysis

Table S12: Relative triple energy contributions ($\Delta\varepsilon_{ijk}$ in E_h) for reactants (R), transition state (TS), and products (P) of system 130 of the pericycle reaction set, showing the 50 triples contributions with the largest deviation energy between SL and ML2 (descending order) which were not sorted out by the pair-selection procedure. The corresponding structure (R, TS, P) and the orbital triple indices ijk of the triples are listed. NormalPNO settings and an aug-cc-pVTZ basis are used.

System	ijk	$\lvert\Delta\varepsilon_{ijk}\rvert$	ε_{ijk} (single-level)	ε_{ijk} (multi-level2)
TS	60, 59, 51	6.24e-04	−2.36e-05	−6.48e-04
R	60, 59, 51	5.98e-04	−1.83e-05	−6.16e-04
TS	60, 59, 28	4.69e-04	−2.07e-05	−4.90e-04
R	67, 58, 37	3.83e-04	−6.12e-06	−3.89e-04
R	59, 51, 34	3.58e-04	−1.31e-05	−3.71e-04
TS	60, 34, 28	3.36e-04	−1.14e-05	−3.47e-04
R	59, 51, 35	3.05e-04	−1.68e-05	−3.22e-04
R	67, 62, 58	2.95e-04	−1.43e-05	−3.09e-04
TS	60, 35, 28	2.92e-04	−1.22e-05	−3.04e-04
TS	60, 41, 28	2.76e-04	−1.10e-05	−2.87e-04
R	67, 59, 51	2.65e-04	−1.12e-05	−2.76e-04
TS	60, 51, 34	2.56e-04	−1.35e-05	−2.70e-04
TS	67, 60, 28	2.48e-04	−1.74e-05	−2.66e-04
TS	60, 38, 28	2.43e-04	−1.09e-05	−2.53e-04
TS	60, 51, 28	2.34e-04	−5.56e-06	−2.40e-04
TS	60, 47, 28	2.32e-04	−5.04e-06	−2.37e-04
R	67, 58, 20	2.32e-04	−1.19e-05	−2.44e-04
R	67, 58, 33	2.20e-04	−1.78e-06	−2.21e-04
TS	60, 59, 47	2.19e-04	−8.84e-06	−2.28e-04
R	59, 51, 51	2.08e-04	−8.38e-06	−2.17e-04
TS	60, 51, 35	1.94e-04	−1.37e-05	−2.07e-04

TS	60, 51, 41	1.86e-04	−1.15e-05	−1.97e-04
TS	60, 28, 28	1.81e-04	−8.04e-06	−1.89e-04
R	59, 59, 51	1.70e-04	−1.41e-05	−1.84e-04
TS	67, 60, 47	1.68e-04	−8.85e-06	−1.76e-04
TS	59, 51, 34	1.67e-04	−1.28e-05	−1.80e-04
TS	60, 47, 34	1.60e-04	−4.45e-06	−1.64e-04
R	59, 51, 28	1.53e-04	−5.65e-06	−1.59e-04
TS	60, 51, 51	1.49e-04	−7.49e-06	−1.57e-04
TS	67, 60, 51	1.35e-04	−7.56e-06	−1.43e-04
TS	59, 34, 28	1.31e-04	−1.01e-05	−1.41e-04
P	51, 41, 22	1.30e-04	−2.40e-05	−1.54e-04
R	67, 58, 58	1.29e-04	−1.42e-05	−1.44e-04
R	59, 51, 44	1.28e-04	−4.37e-06	−1.32e-04
TS	60, 47, 38	1.26e-04	−9.33e-06	−1.35e-04
TS	67, 59, 28	1.25e-04	−1.93e-05	−1.44e-04
R	59, 51, 31	1.24e-04	−4.32e-06	−1.29e-04
TS	60, 60, 51	1.22e-04	−1.29e-05	−1.35e-04
R	67, 58, 54	1.20e-04	−1.89e-06	−1.22e-04
TS	60, 60, 28	1.19e-04	−1.34e-05	−1.33e-04
TS	59, 35, 28	1.19e-04	−1.20e-05	−1.31e-04
R	67, 59, 47	1.17e-04	−1.14e-05	−1.28e-04
R	60, 59, 47	1.17e-04	−7.72e-06	−1.24e-04
TS	59, 51, 35	1.15e-04	−1.43e-05	−1.30e-04
TS	59, 41, 28	1.13e-04	−1.46e-05	−1.28e-04
R	61, 59, 51	1.12e-04	−3.96e-06	−1.16e-04
TS	59, 51, 41	1.11e-04	−1.57e-05	−1.27e-04
TS	60, 47, 47	1.10e-04	−4.75e-06	−1.15e-04

TS	59, 51, 28	1.08e-04	−6.05e-06	−1.14e-04
R	59, 47, 34	1.03e-04	−4.47e-06	−1.08e-04

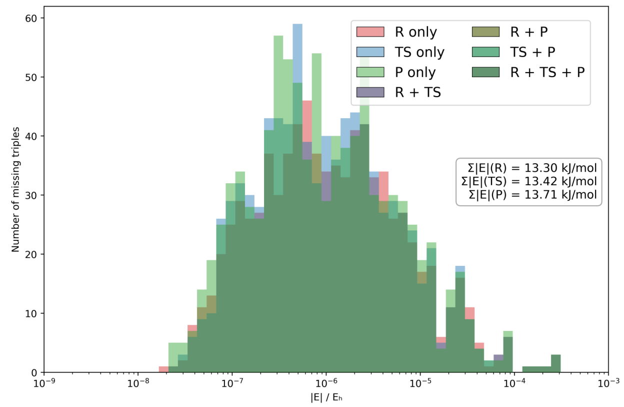

Figure S6: Absolute deviation of missing triples contributions between ML2 and SL DLPNO-CCSD(T_0) calculations for the reactant (R, red), transition state (TS, blue) and product (P, green) of the pericyclic reaction with index 130. The energy is given in E_h and the scale is logarithmic. The aug-cc-pVTZ basis set and NORMALPNO settings are used. The sum of all missing triples contributions is listed on the right for R, TS and P in kJ/mol.

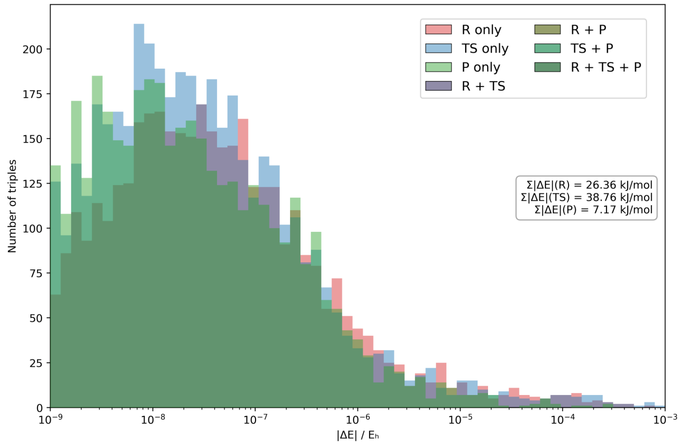

Figure S7: Difference triples energy contributions between ML2 and SL DLPNO-CCSD(T_0) calculations for the reactant (R, red), transition state (TS, blue) and product (P, green) of the pericyclic with the reaction index 130. The energy is given in E_h and the scale is logarithmic. The aug-cc-pVTZ basis set and NORMALPNO settings are used. The sum of all difference triples contributions is listed on the right for R, TS and P in kJ/mol. The thresholds used are $\tau^{\Delta}_{\text{cutPairs}}$ and $\tau^{\Delta}_{\text{cutTriples}}$.

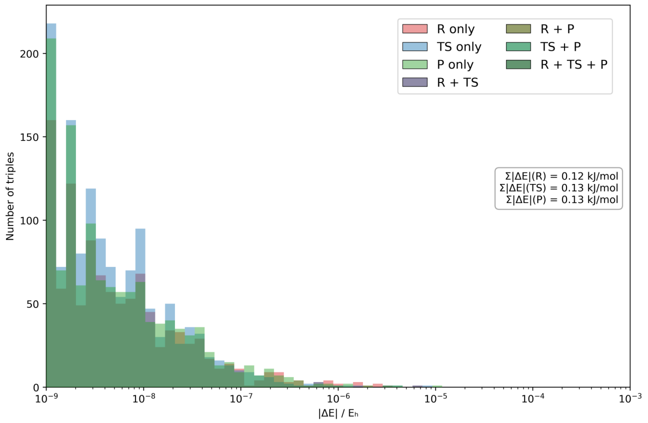

Figure S8: Absolute deviation of triple contributions between ML2 and SL DLPNO-CCSD(T_0) calculations for the reactant (R, red), transition state (TS, blue) and product (P, green) of the pericyclic reaction with index 130. The energy is given in E_h and the scale is logarithmic. The aug-cc-pVTZ basis set and NORMALPNO settings are used. The sum of all difference triples contributions is listed on the right for R, TS and P in kJ/mol. The only multi-level threshold used is $\tau^{\Delta}_{\text{CutTriples}}$.

Table S13: Comparison between triples contributions for reaction energies and barrier heights for pericyclic reactions with the indices 88, 130, 131 and 133. Four different DLPNO-CCSD(T_0) calculations are compared, with aug-cc-pVTZ as basis set and NORMALPNO settings, except for the entry SL-LOOSE (LOOSEPNO is used). ML2 with standard cutoffs and ML2$_{cut}$ with selective cutoffs are compared. The selected cutoffs are: $\tau^{\Delta}_{\text{CutPNOs}}$ and $\tau^{\Delta}_{\text{CutTriples}}$.

Index	Reaction energies				Barrier heights			
	SL-LOOSE	SL-NORMAL	ML2	ML2$_{\text{cut}}$	SL-LOOSE	SL-NORMAL	ML2	ML2$_{\text{cut}}$
88	−0.35	3.38	2.91	−0.77	−37.60	−21.56	8.05	−17.42
130	−2.51	−4.39	12.16	−3.46	−24.95	−27.93	−30.10	−27.24
131	−1.91	−3.78	−11.67	−8.97	−66.27	−27.08	−27.06	−26.53
133	119.74	−3.67	−6.86	−3.28	46.57	−27.23	−22.56	−26.81

References

(1) Prasad, V. K.; Pei, Z.; Edelmann, S.; Otero-de-la Roza, A.; DiLabio, G. A. BH9, a New Comprehensive Benchmark Data Set for Barrier Heights and Reaction Energies: Assessment of Density Functional Approximations and Basis Set Incompleteness Potentials. *J. Chem. Theory Comput.* **2022**, *18*, 151–166.
